# Tissue-resident-like CD4^+^ T cells secreting IL-17 control *Mycobacterium tuberculosis* in the human lung

**DOI:** 10.1172/JCI142014

**Published:** 2021-05-17

**Authors:** Paul Ogongo, Liku B. Tezera, Amanda Ardain, Shepherd Nhamoyebonde, Duran Ramsuran, Alveera Singh, Abigail Ng’oepe, Farina Karim, Taryn Naidoo, Khadija Khan, Kaylesh J. Dullabh, Michael Fehlings, Boon Heng Lee, Alessandra Nardin, Cecilia S. Lindestam Arlehamn, Alessandro Sette, Samuel M. Behar, Adrie J.C. Steyn, Rajhmun Madansein, Henrik N. Kløverpris, Paul T. Elkington, Alasdair Leslie

**Affiliations:** 1 Africa Health Research Institute, Durban, South Africa.; 2 School of Laboratory Medicine and Medical Sciences, University of KwaZulu-Natal, Durban, South Africa.; 3 Institute of Primate Research, National Museums of Kenya, Nairobi, Kenya.; 4 National Institute for Health Research Southampton Biomedical Research Centre, School of Clinical and Experimental Sciences, Faculty of Medicine, and; 5 Institute for Life Sciences, University of Southampton, Southampton, United Kingdom.; 6 Division of Infection and Immunity, University College London, London, United Kingdom.; 7 Department of Cardiothoracic Surgery, Nelson Mandela School of Medicine, University of KwaZulu-Natal, Durban, South Africa.; 8 ImmunoScape Pte Ltd, Singapore, Singapore.; 9 La Jolla Institute for Immunology, La Jolla, California, USA.; 10 Department of Medicine, University of California, San Diego, La Jolla, California, USA.; 11 Department of Microbiology and Physiological Systems, University of Massachusetts Medical School, Worcester, Massachusetts, USA.; 12 Department of Microbiology and; 13 Center for AIDS Research and Center for Free Radical Biology, University of Alabama at Birmingham, Birmingham, Alabama, USA.; 14 Department of Immunology and Microbiology, University of Copenhagen, Copenhagen, Denmark.

**Keywords:** Immunology, Infectious disease, Bacterial infections, T cells

## Abstract

T cell immunity is essential for the control of tuberculosis (TB), an important disease of the lung, and is generally studied in humans using peripheral blood cells. Mounting evidence, however, indicates that tissue-resident memory T cells (Trms) are superior at controlling many pathogens, including *Mycobacterium tuberculosis* (*M*. *tuberculosis*), and can be quite different from those in circulation. Using freshly resected lung tissue, from individuals with active or previous TB, we identified distinct CD4^+^ and CD8^+^ Trm-like clusters within TB-diseased lung tissue that were functional and enriched for IL-17–producing cells. *M*. *tuberculosis*–specific CD4^+^ T cells producing TNF-α, IL-2, and IL-17 were highly expanded in the lung compared with matched blood samples, in which IL-17^+^ cells were largely absent. Strikingly, the frequency of *M*. *tuberculosis*–specific lung T cells making IL-17, but not other cytokines, inversely correlated with the plasma IL-1β levels, suggesting a potential link with disease severity. Using a human granuloma model, we showed the addition of either exogenous IL-17 or IL-2 enhanced immune control of *M*. *tuberculosis* and was associated with increased NO production. Taken together, these data support an important role for *M*. *tuberculosis*–specific Trm-like, IL-17–producing cells in the immune control of *M*. *tuberculosis* in the human lung.

## Introduction

Tuberculosis (TB), caused by the bacterium *Mycobacterium tuberculosis* (*M*. *tuberculosis*), remains a leading cause of death from infectious disease worldwide (1). Despite the availability of anti-TB treatment, difficulties in diagnosis and the emergence of drug resistance warrant the need for new vaccine strategies for TB in humans (2). Approximately 25% of the global population is estimated to have been infected with *M*. *tuberculosis* (3), of which the majority of cases are contained by the immune system, and only 5%–10% progress to active TB during their lifetime (4). The existence of protective immunity in the majority of participants gives hope that the only current vaccine in use, *Mycobacterium bovis* Bacille Calmette-Guérin (BCG), can be improved upon, as this protects infants from disseminated TB but does not provide reliable protection in adults (2).

T cell–based vaccines are promising candidates for TB (5–8), as these cells are fundamental in the prevention of primary disease after initial infection and the development of post-primary TB after latent infection (9–11). However, the vast majority of studies seeking to identify suitable T cell correlates of protection in human blood, which is necessary for effective vaccine design, have failed (8). One possible reason for this is that TB is primarily a disease of the lung, and parabiotic mouse experiments show that most memory T cells in lung tissue do not recirculate in blood (12, 13). Moreover, the adoptive transfer of lung T cells from infected mice provides better protection against *M*. *tuberculosis* challenge than T cells from the blood of the same animals (14), suggesting that lung-resident T cells may be functionally different from those in circulation.

When naive T cells encounter their cognate antigen, they proliferate and differentiate into effector memory T cells, which mount a rapid immune response to the same antigen following reexposure, a process that underlies the basic principles of vaccine intervention (15–17). This memory response is often most potent at the site of infection, where a subset of non-recirculating, rapidly responding memory T cells, called tissue-resident memory T cells (Trms), reside (14). Trms share functional and transcriptional similarities with central and effector memory T cells but persist within tissues for extended periods, largely without recirculation back to blood (18). Because of their positioning and this rapid response time, Trms play critical roles in clearance of different infections at mucosal surfaces (19–23). This important subset is transcriptionally, phenotypically, and functionally distinct from circulating T cells (21, 24) but remains poorly characterized in humans.

Evidence for the importance of lung Trms in the response to *M*. *tuberculosis* comes primarily from experimental animal models, in which non-recirculating T cells in the lung are associated with enhanced protection (25). In addition, in some but not all cases, mucosal vaccination has been shown to offer superior protection than either subcutaneous or intradermal delivery (26–29), which has been linked to the induction of stronger Trm responses. Vaccine-specific Trm responses are associated with reduced bacterial loads, lung pathology, and dissemination, and importantly, provide protection independent of circulating memory T cells (28–30). However, our understanding of the lung Trm response to TB in humans is, understandably, much less complete.

To our knowledge, this study is the first to examine *M*. *tuberculosis*–specific lung Trms from humans. Using a unique cohort of study participants undergoing surgical lung resections as treatment for ongoing or previous TB infection, we characterize T cells isolated from lung tissues in comparison with matched blood from the same donor and find they express Trm markers, are highly functional, are enriched for IL-17–producing subsets, and are partially impaired by HIV coinfection. In addition, we find *M*. *tuberculosis*–specific CD4^+^ T cells are highly enriched in the lung, including TB-specific IL-17^+^ cells, which are largely absent from the blood. Finally, we present ex vivo and in vitro evidence that the IL-17 response is important in immune control of *M*. *tuberculosis* in humans.

## Results

### TB-infected human lung tissue contains functional effector memory T cells expressing tissue-resident markers.

**Figure 1:**
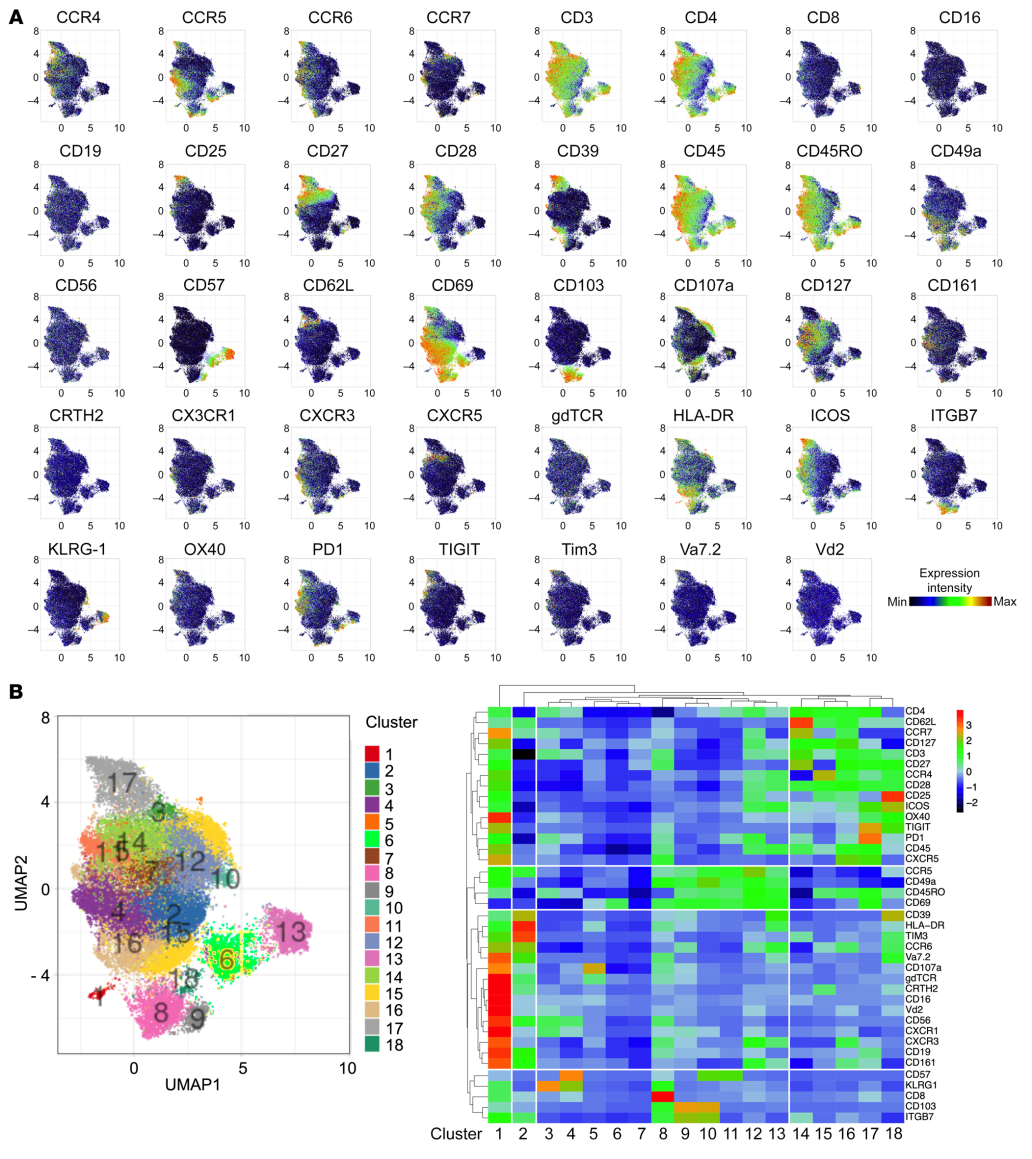
Human lung tissue contains populations of Trm-like T cells. (**A**) Cumulative staining of lung CD4^+^ T cells from 12 biological replicates, defined as having either active TB or previous TB, by CyTOF high-dimensional phenotyping based on uniform manifold approximation and projection (UMAP) plotted as UMAP1 (*x* axis) versus UMAP2 (*y* axis) for each cell type. CRTH2, chemoattractant receptor–homologous molecule expressed on receptor on Th2 cells; ITGB7, integrin β7; PD-1, programmed cell death 1; TIGIT, T cell immunoreceptor with immunoglobulin and ITIM domain. (**B**) PhenoGraph clustering (left) identified 18 clusters (clusters 1–18) depicted on the heatmap of staining intensity of T cell markers (right).

**Figure 2:**
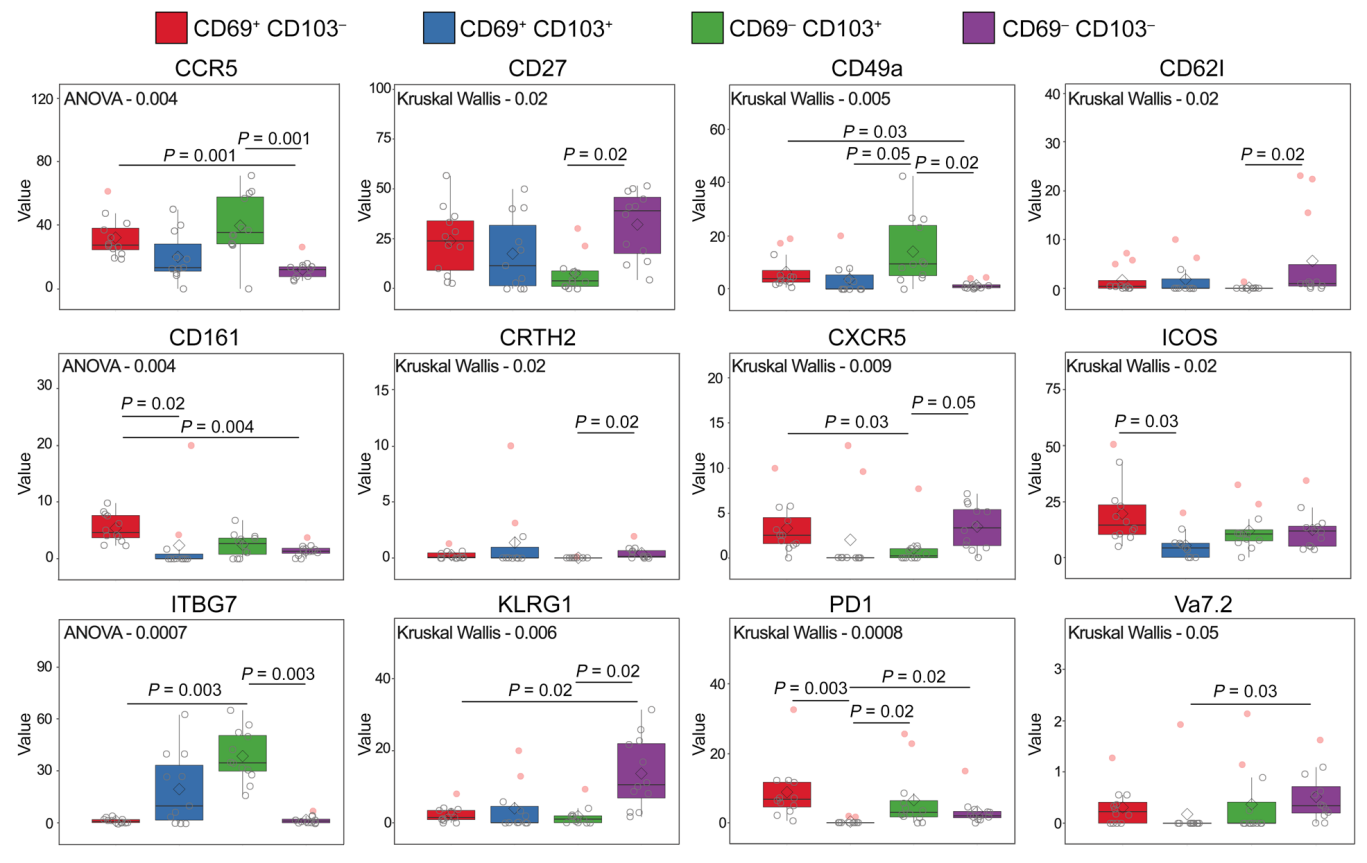
Expression pattern of surface markers in CyTOF panel significantly differentially expressed on CD4^+^ T cells in lung homogenate according to coexpression of CD69 and CD103. Markers not significantly expressed presented in Supplemental Figure 1A. Significance test applied stated within each individual plot.

To examine the T cells present in the TB-infected human lung, we obtained small tissue pieces from surgically resected lungs, removed from participants with active or prior TB disease to treat their TB or post-TB sequelae. Multiple tissue sections from the same lung (typically 3) were homogenized and pooled to generate a single-cell suspension and analyzed by cytometry. In humans, Trms can be differentiated from circulating cells of the lung vasculature based on the expression of several surface markers associated with tissue retention, including expression of CD69 and CD103 and loss of CD62L (13, 14, 31–37). First, we applied high-dimensional cytometry by time of flight (CyTOF) for detailed phenotyping of T cell populations present in lung homogenate. Data presented in Figure 1, A and B, represent the cumulative staining of CD4^+^ T cells from 12 biological replicates, defined as having either active TB or previous TB as discussed below. T-distributed stochastic neighbor embedding analysis revealed distinct clusters of T cells within lung homogenate. As expected, given the perfused nature of lungs, these included both CD69^+/–^ CD4^+^ T cell populations and CD103^+^ clusters expressing high levels of CD69. The same clusters expressing high levels of CD69 were observed for CD8^+^ T cells (Supplemental Figure 2, A and B; supplemental material available online with this article; https://doi.org/10.1172/JCI142014DS1). Likewise, the great majority of CD103^+^ cells costain with ITGB7, which forms a heterodimer with CD103 to make the tissue residency integrin αEβ7, a ligand for E-cadherin. Conversely, CD62L expression is largely confined to CD69^–^ clusters, as is KLRG1, a marker found to be expressed by T cells in lung vasculature but not in the parenchyma of *M*. *tuberculosis–*infected mice (14). Other Trm-like clusters of note include cluster 11 — CD69^+^ cells that coexpress PD-1, TIGIT, CXCR5, and ICOS, consistent with the phenotypic description of lung-resident T follicular helper cells (38) that were shown to be important in the mouse model of TB (39) — and cluster 16 — CD69^+^ cells that coexpress CCR6 and CCR4, classical markers of Th17 cells (40), and CD161 and CD39, novel Th17 markers (41, 42). In addition to this analysis, we grouped cells according to expression of CD69 and CD103 and tested expression levels of the other markers used. For CD4^+^ and CD8^+^ T cells, 12/32 and 18/32 surface markers, respectively, were differentially expressed in association with CD69 and/or CD103, including upregulation of several markers associated with tissue residency in other studies, such as CCR5, CCR6, CD49a, CXCR3, and CD161 (refs. 25, 43, 44; Figure 2; and Supplemental Figure 1, Supplemental Figure 2, and Supplemental Figure 3).

**Figure 3:**
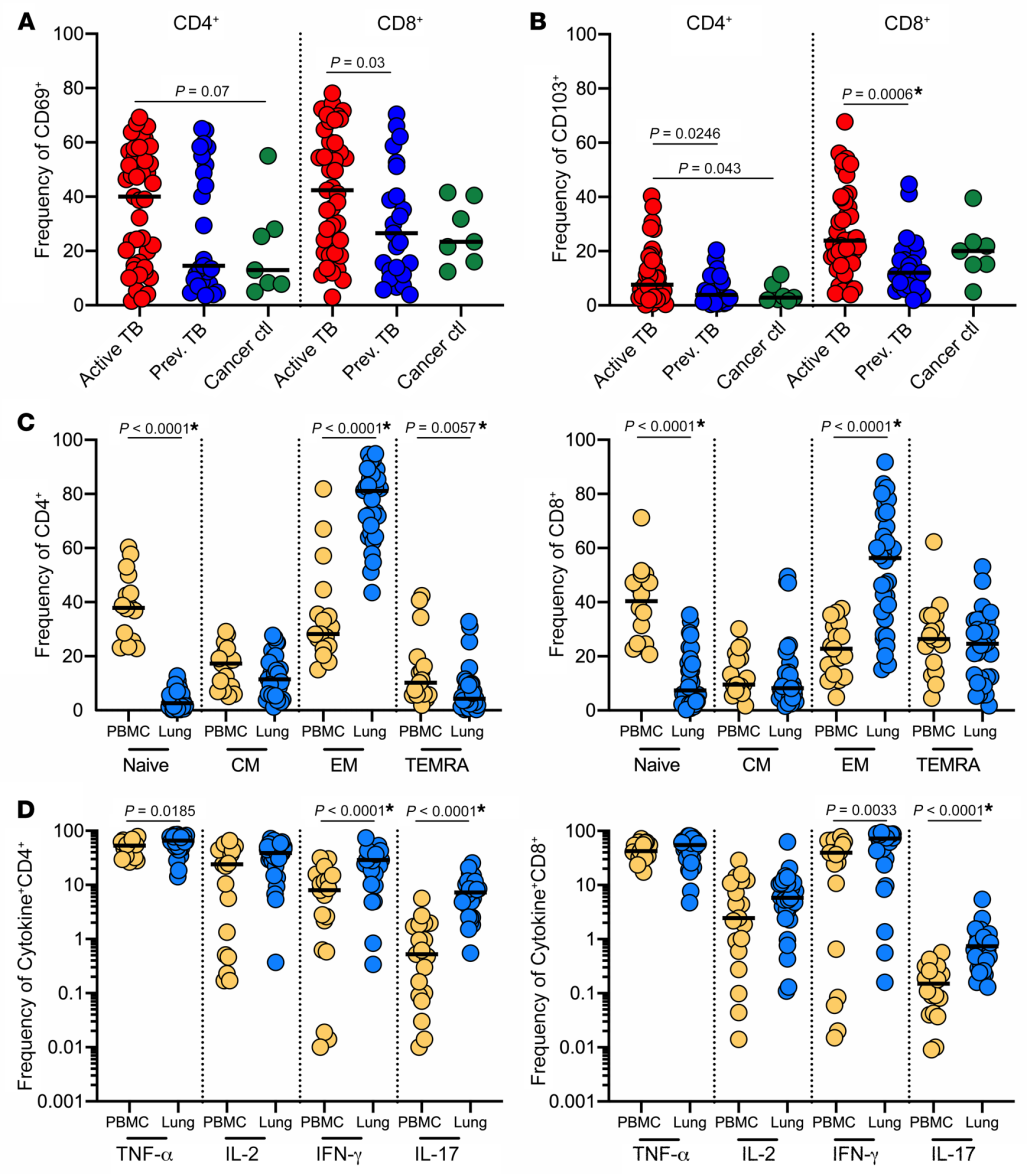
Lung Trm-like T cells are functional and predominantly effector memory. (**A**) Frequencies of CD69^+^ CD4^+^ and CD8^+^ T cells isolated from lung tissues from participants with active TB (red), participants with previous TB (dark blue), or cancer controls (dark green). (**B**) Frequencies of CD103^+^ CD4^+^ and CD8^+^ T cells isolated from lung tissues from participants with active TB (red), participants with previous TB (dark blue), or cancer controls (dark green). (**C**) Frequencies of CD4^+^ (left) and CD8^+^ T cells (right) expressing naive, central memory (CM), effector memory (EM), and terminally differentiated effector memory T cells (TEMRA) phenotypes in blood (yellow) and lungs (blue) from participants with active/previous TB. (**D**) Frequencies of TNF-α–, IL-2–, IFN-γ–, and IL-17–producing CD4^+^ and CD8^+^ T cells from blood (yellow) and lungs (blue) of participants with active/previous TB. Significance calculated by Mann-Whitney test. Asterisk denotes *P* values that remained significant after stringent Bonferroni’s correction for multiple comparisons.

Having shown that CD69^+^ and CD103^+^ cells in human lungs express other phenotypic markers consistent with Trms, we examined the expression of these markers by flow cytometry in a larger cohort and demonstrated enrichment of CD69 and CD103 relative to the circulation and reduced expression of CD62L (Supplemental Figure 4, A and B). Next, to investigate the impact of *M*. *tuberculosis* infection, we quantified these subsets in participants with suspected active TB or previous pulmonary TB, as determined by the operating surgeon, based on clinical history, presentation, and preoperative chest x-ray/CT scan. Macroscopically uninvolved tissue margins from participants undergoing lung cancer resection were used as non-TB controls. Microbiological confirmation was not consistently available. Therefore, the treating clinician classified patients into groups at the time of surgery, and clinicians were not involved in the subsequent analysis. Based on this, individuals in the active TB group were enriched for CD4^+^ and CD8^+^ cells expressing CD69 compared with previous TB or cancer controls, reaching significance for CD8^+^ T cells (Figure 3A). The apparent bimodal distribution of CD69^+^ frequency was correlated to clinical characteristics, but no clear pattern was identified, although this may reflect a lack of statistical power in this group size. Similarly, the frequency of CD103^+^ CD4^+^ and CD8^+^ T cells was significantly higher in the active TB group (Figure 3B). As reported previously, CD8^+^ T cells in the lung expressed higher levels of CD103 than CD4^+^ T cells. Approximately 50% of T cells in lung homogenate expressed a combination of CD69 and CD103, although the relative proportions varied between participants (Supplemental Figure 5, A–C). In contrast, expression of these markers was extremely low in matched blood samples and was not significantly different between patient groups (Supplemental Figure 5D). Thus, these data suggest that CD4^+^ and CD8^+^ Trm-like cells are present in TB-diseased human lung tissue and are likely to have expanded during active TB infection.

In addition to expression of cell surface markers such as CD69, long-lived Trm cells may also be distinguished by their memory phenotype (45). Using CD45RA and CCR7 to distinguish naive (CD45RA^+^CCR7^+^), effector memory (CD45RA^–^CCR7^–^), central memory (CD45RA^–^CCR7^+^), and terminally differentiated effector memory (TEMRA) (CD45RA^+^CCR7^–^) T cells, we found *M*. *tuberculosis*–infected human lungs were highly enriched for effector memory CD4^+^ and CD8^+^ T cells, compared with predominantly naive cells in matched blood (Figure 3C). Moreover, effector memory cells were highest in the CD69^+^ fraction for both CD4^+^ and CD8^+^ T cells, while naive and TEMRA cells were significantly lower (Supplemental Figure 5E). The same was true for CD103^+^ CD8^+^ but not CD4^+^CD103^+^ T cells. Strikingly, effector memory T cells were also highly enriched in the CD69^–^ fraction compared with naive cells, which may suggest the presence of Trms in lung tissue that lack CD69, as demonstrated in the mouse model (46).

Next, to assess T cell functionality, lung homogenate and matched blood samples were nonspecifically stimulated with mitogen (PMA and ionomycin), and cytokine production was measured by intracellular cytokine staining. Consistent with an enrichment of effector memory cells, a greater proportion of CD4^+^ T cells isolated from lung tissue produced TNF-α, IFN-γ, and IL-17 than cells in circulation, while lung CD8^+^ T cells produced more IFN-γ and IL-17 (Figure 3D; data presented on a log scale). In both cases, the increased frequency of IL-17–producing T cells was the most striking (14.6- and 4.7-fold increase for CD4^+^ and CD8^+^ T cells, respectively), in line with described enrichment of Th17 and Tc17 subsets within the lung (47–49). To examine this in more detail, we sought to examine the frequency of cytokine-producing cells coexpressing CD69 and CD103. However, the mitogenic stimulation approach used in this study resulted in the upregulation of CD69, despite bypassing the TCR (Supplemental Figure 6A and ref. 50), making CD69 expression data difficult to interpret on stimulated cells. CD103 expression, in contrast, was not affected by PMA and ionomycin stimulation (Supplemental Figure 6B). The CD103^+^ fraction contained the highest frequency of cytokine-producing cells, particularly of IFN-γ and IL-17, of which IL-17 was almost exclusively produced by the CD103^+^ subset (Supplemental Figure 6, C and D). This trend was reduced in CD8^+^ T cells, although IL-17–producing CD8^+^ T cells were also predominantly CD103^+^. Overall, these data show that TB-diseased human lung tissue contains T cells that upregulate surface markers of Trms, are highly enriched for memory subsets, and are highly functional and enriched for IL-17–producing subsets.

### HIV infection is associated with decreased functionality in tissue-resident T cells from TB-infected lungs.

HIV coinfection rates were high among study participants, and although all participants were taking antiretroviral therapy (ART) at the time of surgery, treated HIV remains an important independent risk factor for TB (51). Therefore, the effect of HIV on lung-associated T cells was evaluated using CD4/CD8 ratio (52). In blood from healthy controls, a median CD4/CD8 ratio of 2.04 was observed, within the expected range of 1.5–2.5 for healthy humans (53). However, HIV-infected participants displayed a reduced CD4/CD8 ratio in blood (median 0.93, *P* = 0.004 vs. healthy controls) (Supplemental Figure 7A), suggesting persistent immunodeficiency despite ART. In the lung, HIV^–^ participants and cancer controls in our study displayed a median ratio of 1, lower than the expected value of 2 (31), suggesting immune perturbation in these participants. However, HIV coinfection was associated with highly significant further reduction of the lung CD4/CD8 ratio (0.5, *P* < 0.0001 vs. HIV^–^ participants), indicating an additional profound impact of HIV infection on lung T cells in these diseased participants (Supplemental Figure 7B).

**Figure 4:**
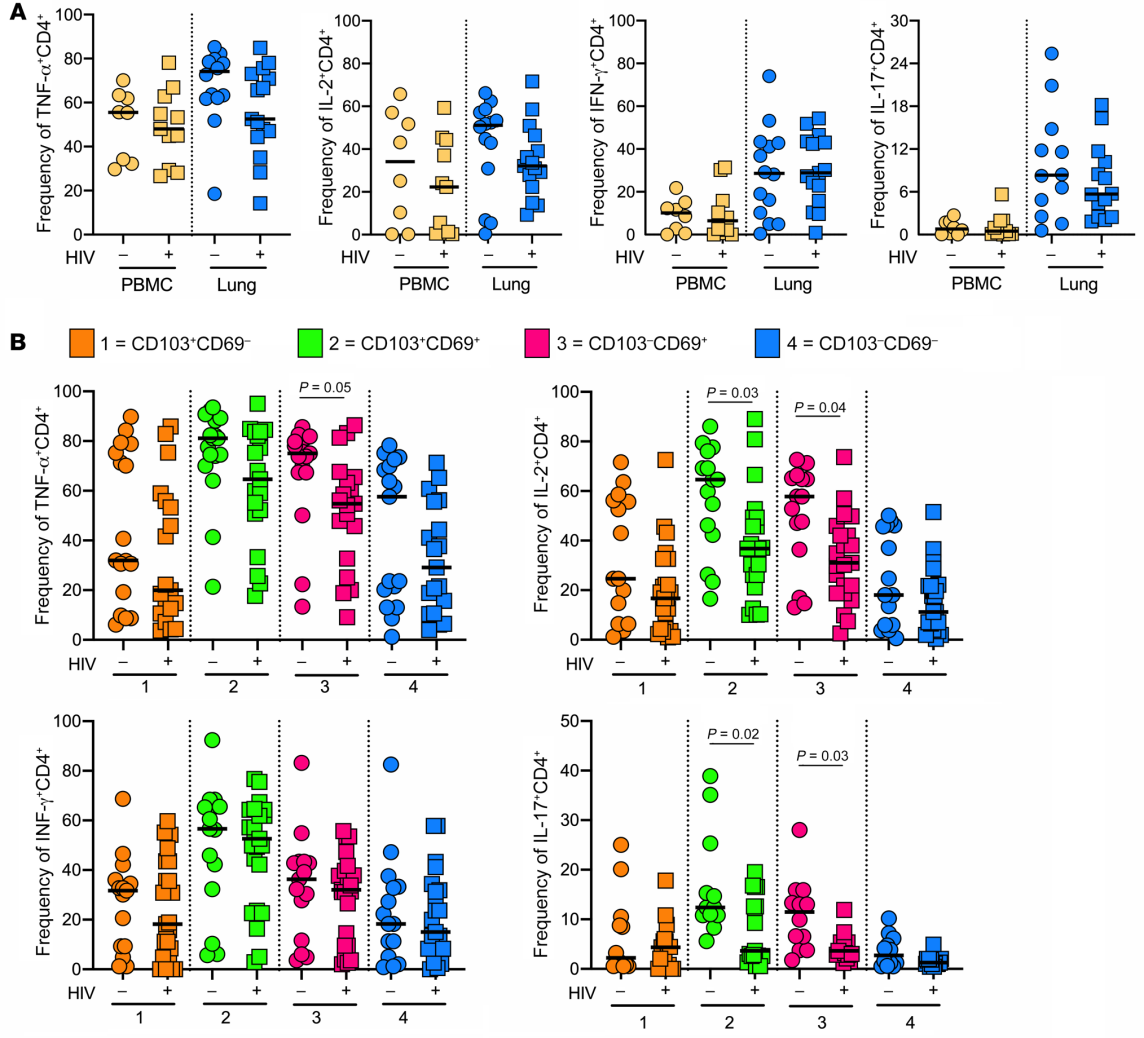
HIV severely depletes cytokine-producing T cells from the lungs of TB-infected study participants. (**A**) Frequencies of TNF-α–, IL-2–, IFN-γ–, and IL-17–producing CD4^+^ T cells from blood (yellow) and lungs (blue) of participants with (squares) and without (circles) HIV coinfection. (**B**) Tissue-resident phenotypes of TNF-α–, IL-2–, IFN-γ–, and IL-17–producing CD4^+^ T cells from lungs from participants with (squares) and without (circles) HIV coinfections, where 1 (orange) = CD103^+^CD69^–^, 2 (green) = CD103^–^CD69^+^, 3 (pink) = CD103^+^CD69^+^, and 4 (blue) = CD103^–^CD69^–^. Significance calculated by Mann-Whitney test.

Surprisingly, HIV infection was not associated with a significant decrease in the frequency of cytokine-producing cells in either bulk lung CD4^+^ T cells or matched PBMCs (Figure 4A). However, when we examined cytokine production according to expression of tissue residency markers within the lung, the frequencies of CD103^–^CD69^+^ CD4^+^ T cells producing TNF-α, IL-2, and IL-17 and CD103^+^CD69^+^ CD4^+^ T cells producing IL-2 and IL-17 were significantly lower in HIV-infected individuals compared with HIV-uninfected individuals (Figure 4B). Similarly, no differences were observed in blood of bulk lung CD8^+^ T cells; however, only the frequency of TNF-α–producing CD8^+^ T cells was significantly reduced in the fraction expressing tissue-resident markers (Supplemental Figure 8, A and B). Overall, these data show that HIV coinfection leads to persistent skewing of CD4/CD8 ratio in the lung and a deficit in lung T cell functionality, most notably in IL-2 and IL-17 production.

### TB-specific T cells are enriched in the lung and predominantly CD103^–^ tissue-resident effector memory cells.

**Figure 5:**
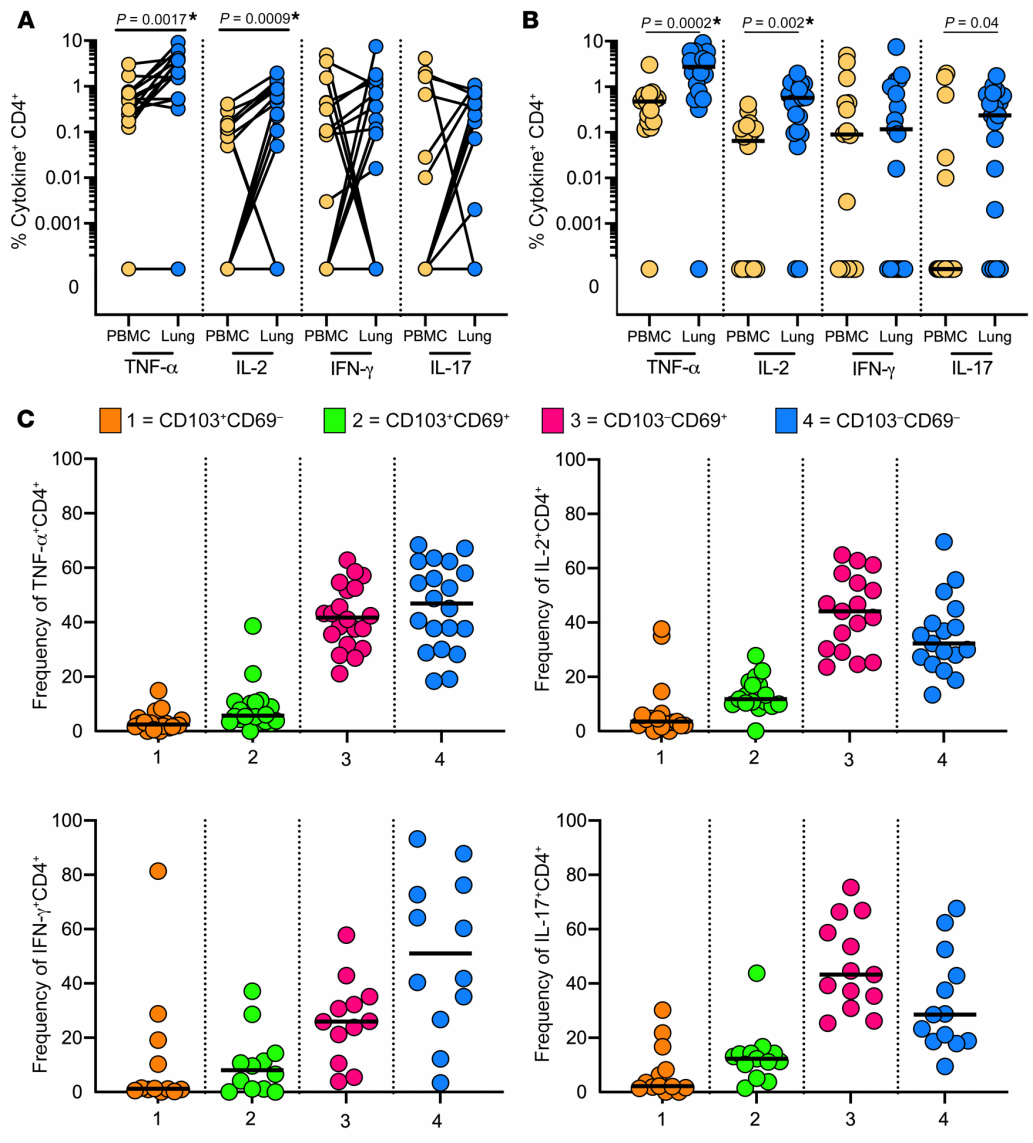
A portion of lung T cells are TB specific and produce cytokines in response to *M. tuberculosis* peptides. (**A**) Frequencies of TB-specific TNF-α–, IL-2–, IFN-γ–, and IL-17–producing CD4^+^ T cells in paired blood (yellow) and lung (blue) samples from the same participant. Significance by Wilcoxon’s matched pairs signed-rank test. (**B**) Frequencies of TB-specific TNF-α–, IL-2–, IFN-γ–, and IL-17–producing CD4^+^ T cells in blood (yellow) and lung (blue) samples from participants with active/previous TB. Significance by Mann-Whitney test. Asterisk denotes *P* values that remained significant after stringent Bonferroni’s correction for multiple comparisons. (**C**) Tissue-resident phenotypes of TNF-α–, IL-2–, IFN-γ–, and IL-17–producing CD4^+^ T cells where 1 (orange) = CD103^+^CD69^–^, 2 (green) = CD103^–^CD69^+^, 3 (pink) = CD103^+^CD69^+^, and 4 (blue) = CD103^–^CD69^–^. Significance calculated by Kruskal-Wallis test, although none was found.

Having determined that T cells isolated from TB-diseased lungs overall retained functionality in terms of cytokine production, we next assessed their responsiveness to TB-specific antigens, using MTB300, a peptide pool of 300 *M*. *tuberculosis* epitopes (54). We compared paired blood and lung samples when available (Figure 5A), in addition to all lung samples (Figure 5B). In the blood, MTB300-specific responses were detected in 16/17 participants, all of which produced TNF-α (median frequency 0.48% of CD4^+^ T cells), and a proportion of which also had detectable, but less frequent, IFN-γ– and IL-2–producing cells (median frequency 0.08% in 10/17 participants and 0.05% in 9/17 participants, respectively). IL-17 production was detected in only 6/17 participants analyzed (Figure 5B). Using the same assay, MTB300-specific CD4^+^ T cells in matched lung samples were significantly higher, with an approximately 5-fold enrichment of TNF-α–producing cells relative to blood (2.39% vs. 0.48%, *P* = 0.002) and a 10-fold enrichment in IL-2 (0.5 vs. 0.05%, *P* < 0.001; Figure 5A). Surprisingly, MTB300-specific cells producing IFN-γ cells were not enriched in the lung. However, MTB300-specific IL-17^+^ cells did appear to be enriched in the lung. This difference became significant when all lung samples were considered (Figure 5B, *P* = 0.04). Moreover, a greater proportion of lung samples contained detectable IL-17 responses compared with the blood (15/19 vs. 5/16, *P* = 0.007 by Fisher’s exact test). It is important to note that, as above, the data are presented on a log scale, and IL-17–producing T cells are detected at a lower frequency than those making TNF-α.

As expected, the majority of cytokine-producing *M*. *tuberculosis*–specific T cells displayed an effector memory phenotype (Supplemental Figure 9). However, despite the fact that CD103^+^ T cells appeared to be the most prolific cytokine producers by nonspecific stimulation (Supplemental Figure 6, C and D), MTB300-responsive cells were almost all CD103^–^, with the most abundant cytokine production observed in the CD103^–^CD69^+^ and CD103^–^CD69^–^ fractions (Figure 5C). Surprisingly, given our earlier findings, although the frequency of TB-specific CD4^+^ T cell responses in HIV^+^ participants tended to be lower than HIV^–^ participants, these differences were not significant (Supplemental Figure 10). In addition to memory markers, T cells were stained for forkhead box P3 (FoxP3) transcription, a canonical marker of regulatory T cells (Tregs). Overall, the lung contained fewer FoxP3^+^ cells than circulation (Supplemental Figure 11A), and the frequency was not increased in HIV-infected participants (Supplemental Figure 11B). However, the frequency of TB-specific CD4^+^ T cells that expressed FoxP3 was over 6-fold higher in the lung compared with the blood (2.53% vs. 0.38%; *P* < 0.0001, Supplemental Figure 11C), with the majority of cells producing TNF-α. Again, these cells lacked CD103 expression, and about 50% expressed CD69 (Supplemental Figure 11D). Although FoxP3 is associated with Tregs, it is also transiently upregulated on activated T cells (55). Therefore, while it is tempting to speculate these data support an expansion of *M*. *tuberculosis*–specific Tregs in the lung, more work is required to confirm this.

### TB-specific IL17^+^ cells correlate with reduced systemic inflammation.

**Figure 6:**
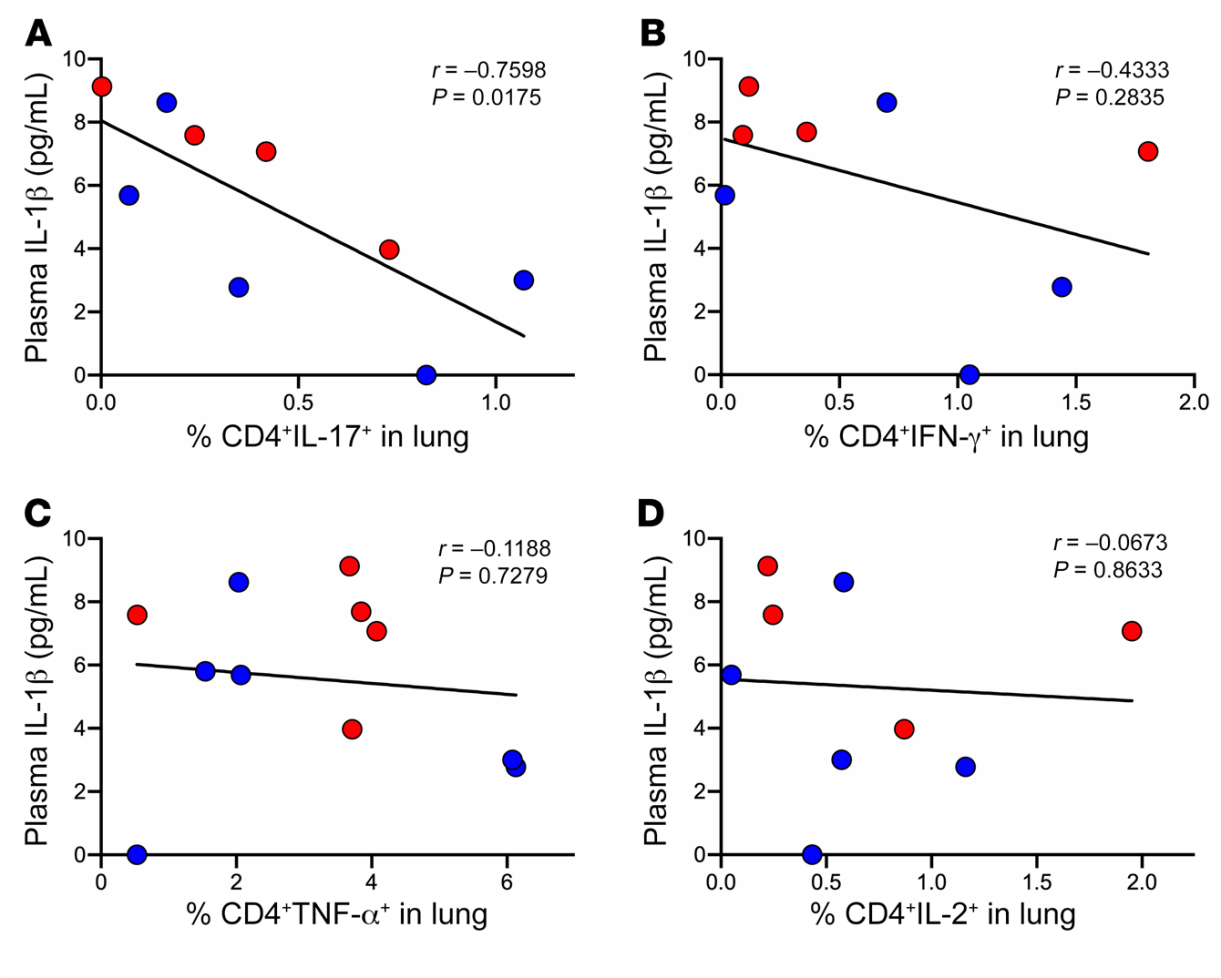
IL-17–producing *M.* *tuberculosis*–specific CD4^+^ T cells in lung homogenate inversely correlate with systemic markers of inflammation. (**A**–**D**) Correlations between plasma IL-1β and TNF-α–, IL-2–, IFN-γ–, and IL-17–producing CD4^+^ T cells in lung tissue from participants with active TB (red) or previous TB (dark blue). Spearman’s correlation with *P* value reported for 2-tailed analysis.

As discussed, the TB-infected participants in this study were categorized as having active TB based on the assessment of the operating surgeon and had varying degrees of disease severity that, for practical reasons, were not precisely defined. Therefore, in a subset of participants, we measured plasma levels of TNF-α, IL-17A, and IL-1β because these proinflammatory cytokines have been shown to directly correlate with TB disease severity in the lung and bacterial burden (56, 57). Both the active and previous TB groups analyzed contained participants with elevated levels of these cytokines compared with non-TB controls (Supplemental Figure 12). However, this was not true of most participants and suggests a range of disease severity. Next, in participants for whom we had both T cell and cytokine data, we tested whether the frequency of *M*. *tuberculosis*–specific T cells was associated with plasma cytokine levels, as a potential indicator of lung disease severity. Surprisingly, we found a significant inverse relationship between the frequency of *M*. *tuberculosis*–specific IL-17^+^ CD4^+^ T cells and plasma IL-1β concentration (Figure 6A; *r* = –0.7598; *P* = 0.0175). Similar trends were observed between TB-specific IL-17^+^ T cell frequency and TNF-α and IL-17A; however, the associations were less strong and not significant (data not shown). Notably, plasma IL-1β did not correlate with the frequency of TB-specific T cells producing IFN-γ, TNF-α, or IL-2 (Figure 6, B–D). Although the numbers of subjects for whom both cytokine and T cell data were available were small, these data suggest that IL-17–producing T cells could be more important in vivo than the other cytokines measured.

### Exogenous IL-17 and IL-2 are protective in a functional 3D granuloma model.

**Figure 7:**
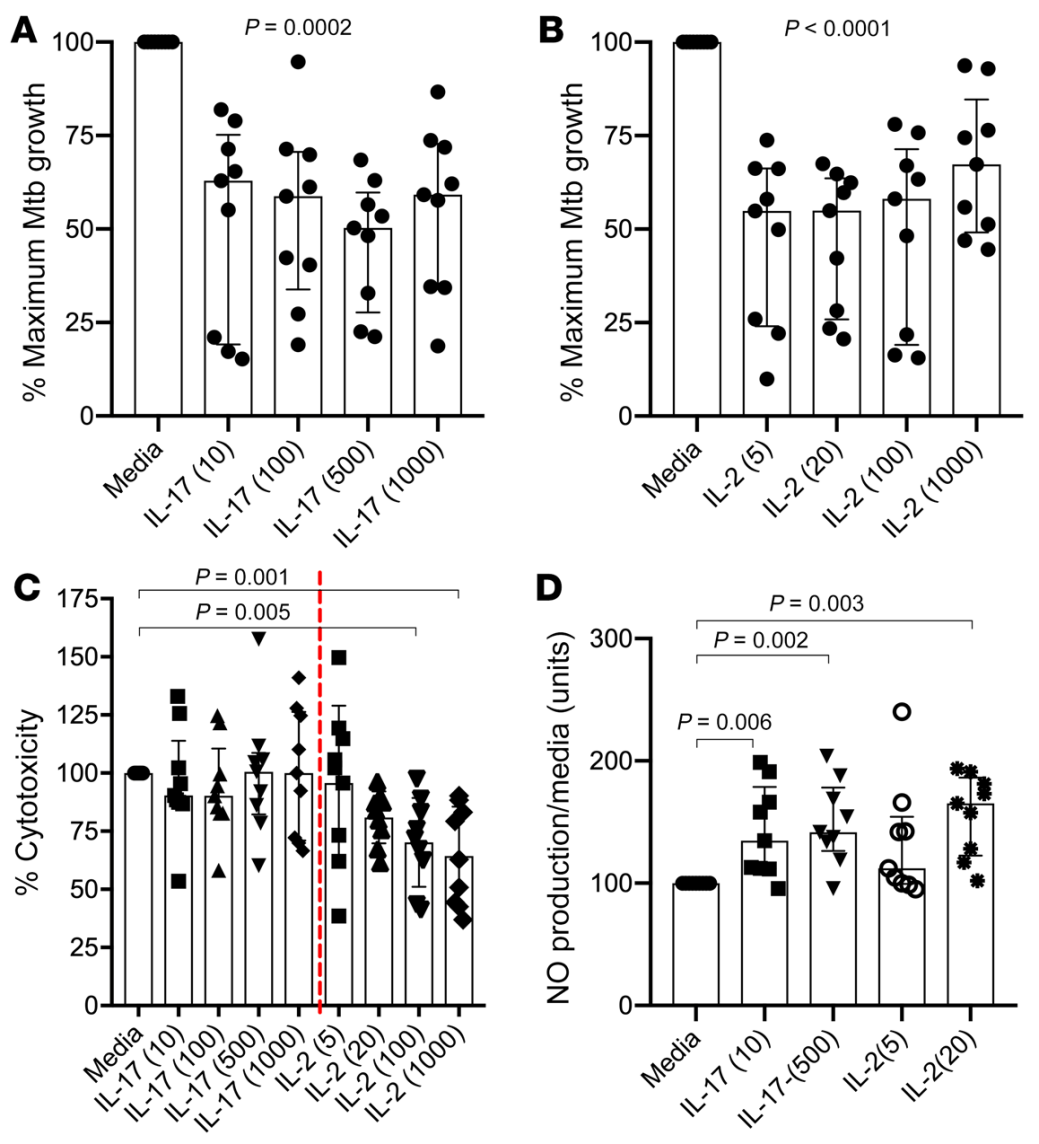
Exogenous IL-17 and IL-2 decrease *M.* *tuberculosis* growth in 3D culture system. (**A** and **B**) Addition of IL-17 and IL-2 to culture media decreases *M*. *tuberculosis* growth in granuloma-like 3D human cell culture system. Data from 3 separate experiments using PBMCs from 3 healthy controls conducted in triplicate. Concentrations shown in parentheses (ng/mL). Impact of exogenous IL-17 and IL-2 on (**C**) cell viability in above experiments as measured by concentration of lactate dehydrogenase (LDH) in culture supernatant on day 7 and (**D**) production of NO. Statistical differences tested by Kruskal-Wallis (**A** and **B**), with Dunn’s correction for multiple comparisons (**C** and **D**).

Finally, to determine whether the T cell subsets identified in the clinical study may improve control of *M*. *tuberculosis*, we studied these different cytokines in a functional granuloma model (58), with the researcher uninformed of the clinical findings. Primary human immune cells were infected with luminescent *M*. *tuberculosis* and then encapsulated in collagen/alginate microspheres, within which granuloma-like structures self-aggregate that recapitulate many features of human lung granuloma (58–60). Microspheres were generated using PBMCs from 3 healthy donors and incubated with TNF-α, IFN-γ, IL-2, or IL-17. Consistent with other published work using this model (58, 61), IFN-γ and TNF-α promoted *M*. *tuberculosis* growth over 12 days compared with controls without cytokines (Supplemental Figure 13A). By contrast, *M*. *tuberculosis* growth was significantly decreased when the microspheres were incubated in media containing either IL-17 or IL-2 (Figure 7, A and B). For both cytokines, we observed no clear titration effect, and there was no clear evidence of synergism when added together (Supplemental Figure 13B). Bacterial growth in the model system is measured by luminescence, and we confirmed the suppressive effect of exogenous IL-17 and IL-2 by CFU on day 15 (Supplemental Figure 13C). Finally, to investigate potential mechanisms of *M*. *tuberculosis* growth inhibition in the microsphere system, we determined the effect of exogenous IL-2 and IL-17 on cell survival and production of NO, an important antimicrobial factor. Exogenous IL-2 but not IL-17 significantly reduced cell death within *M*. *tuberculosis*–infected microspheres, as measured by lactate dehydrogenase (LDH) levels on culture supernatant on day 7 (Figure 7C). Both IL-17 and IL-2 significantly increased NO production at the same time point (Figure 7D).

## Discussion

Multiple studies have demonstrated a crucial protective role for Trms in the immune response to pathogens, and there is an increasing interest in exploiting their potential for improved treatment or vaccine strategies, particularly in TB (28–30). While it is clear that Trms are functionally distinct from circulating T cell populations, to date studies of Trms in TB have been restricted to animal model systems due to the limitations in the availability of fresh human lung tissues. Here, we show that functional cells expressing Trm markers are present in TB-diseased human lung tissue and are highly enriched for IL-17–producing subsets and for *M*. *tuberculosis*–reactive T cells, including *M*. *tuberculosis*–specific IL-17–producing cells. By focusing on Trm-like subsets, we also find that HIV coinfection impairs both IL-17 and IL-2 production from lung-resident T cells. Although we did not find a significant impact of HIV on *M*. *tuberculosis*–specific T cell frequency, these data suggest one mechanism through which HIV increases the risk of developing active TB. The importance of IL-17 in particular is also suggested by the inverse correlation between plasma IL-1β and the frequency of TB-specific IL-17^+^ CD4^+^ T cells. Finally, using a 3D model that mimics aspects of human granuloma, we find that addition of exogenous IL-17 and IL-2 both significantly reduced TB growth (Figure 7), while growth is enhanced by exogenous TNF-α and IFN-γ. Taken together, these data suggest that subsets of *M*. *tuberculosis*–specific T cells are sequestered in the human lung, are functionally different from those detected in circulation, and are likely to play an important role in the immune control of *M*. *tuberculosis* in this tissue.

It is thought that Trms populate specific tissues and become enriched for particular specificities following pathogen exposure. For example, during influenza in mice, Trms are essential for in situ immunity after reexposure (62, 63), and influenza-specific Trms have been identified in human lung parenchyma (64). The potential protection offered by these cells in TB directly was demonstrated by adoptive transfer experiments in mice (14, 65). These studies show that *M*. *tuberculosis* infection leads to the retention of *M*. *tuberculosis*–specific CD4^+^ T cells in the lung, and these cells provide superior control of *M*. *tuberculosis* infection when adoptively transferred into susceptible T cell–deficient hosts compared with blood T cells from the same animal. Although these studies did not examine tissue residency markers, we initially hypothesized that *M*. *tuberculosis*–specific Trms would express CD103, partly because epithelial macrophages in TB granuloma upregulate E-cadherin, the ligand for the integrin formed by CD103 and ITGB7, αEβ7 (66). However, our finding that *M*. *tuberculosis*–specific T cells in our TB lung homogenate were almost exclusively CD103^–^ is consistent with recent work showing that *M*. *tuberculosis* antigen–specific Trms induced by vaccination in the mouse lung express low levels of CD103 (67).

The presence of Th17 Trm-like subsets in TB-infected human lungs is supported by the CyTOF analysis. Phenotypically, Th17 cells are defined by surface expression of CCR6 and CCR4, and *M*. *tuberculosis*–specific T cells in blood that produce IL-17 express these markers (40). CD161 has also been described as a surface marker that distinguishes Th17 expressing the retinoic acid receptor-related orphan receptor γT transcription factor (68), and CD3^+^CD161^+^ IL-17–producing T cells have been detected in human bronchoalveolar lavage fluid (41). In addition, coexpression of CD39 and CD161 has been associated with driving Th17 polarization in the gut mucosa (69), and CD39 expression by Th17 cells was found to enhance their resistance to inflammation-induced cell death through conversion of extracellular ATP (70). Therefore, the distinct CD69^+^ T cell subsets in the lung that coexpress CCR6, CCR4, CD161, and CD39 may represent an important lung Th17 subset. Interestingly, in a mouse model of colitis, this CD39^+^ Th17 subset plays an important role in resolving inflammation through production of IL-10 (70). Overexpression of IL-17 is seen as detrimental to the *M*. *tuberculosis* immune response (71). However, T cell production of IL-10, particularly in combination with IL-17, has been associated with granuloma with no culturable bacteria in nonhuman primates (NHPs) (72). In addition to Th17 cells, T cells that produce both IFN-γ and IL-17 cytokines (Th1* cells) can produce IL-17 in response to *M*. *tuberculosis* antigen (73). We did not distinguish IL-17–producing subsets in our stimulation experiments, and thus either or both Th17 and Th1* cells may be involved. Moreover, CXCR3 is expressed by some cells within the potential Th17 Trm cluster revealed by CyTOF, which is more closely associated with Th1 than Th17 subsets (49). Further work is needed to investigate lung Th17 and Th1* subsets and to explore the potential involvement of additional cytokines, including IL-10. Although we do not find evidence for a protective role for IFN-γ and TNF-α in this study, a wealth of data indicate that complete loss of the cytokines renders both animals and humans highly susceptible to *M*. *tuberculosis* (7). Conversely, elegant studies in mice and NHPs from Dan Barber’s group show that the loss of immune control in the lung associated with interference in the PD-1 immune checkpoint axis is directly related to excessive production of IFN-γ and TNF-α (most recently ref. 74). Thus, although these data and a growing number of studies suggest an important role for IL-17 in the immune response to *M*. *tuberculosis*, it is highly likely that a balanced immune response is still required (75).

The association of treated HIV coinfection with reduced IL-17–producing T cells in the lung is consistent with observations of selective depletion of Th17 from other mucosal compartments, including the gut (76, 77) and female genital tract (78). Failure of ART to restore IL-17–producing T cells in the female genital tract and the gut mucosa, despite successful reconstitution in the blood, has been linked to reduced CCL20 levels for recruiting Th17 via CCR6 (78, 79). However, it is important to note that, in one of the above studies, Th17 cells were found not to be depleted from bronchoalveolar lavage (BAL) fluid of HIV-infected participants (77). Likewise, other groups have described reduced TB-specific CD4^+^ T cells in BAL fluid cells of HIV-coinfected participants compared with noninfected controls (80, 81). In contrast, we did not find a significant reduction in the frequency of TB-specific CD4^+^ lung T cells in HIV-infected participants. More recently, however, the decreased frequency of TB-specific CD4^+^ T cells in BAL fluid of HIV-infected participants was attributed to a large influx of T cells into the BAL fluid (82). When this was accounted for, HIV infection was actually found to have no impact on the absolute number of TB-specific T cells in BAL fluid. These findings suggest challenges in comparing the observations from human BAL fluid and tissue and may be consistent with our observed lack of depletion of *M*. *tuberculosis*–specific CD4^+^ lung T cells in HIV-coinfected participants. The highly skewed CD4/CD8 ratio observed in the lungs of HIV-infected participants is also consistent with data from humanized mice and NHPs showing profound depletion of parenchymal CD4^+^ T cells from the lung (44). This was associated with high expression of the HIV coreceptor, CCR5, which our CyTOF data show is highly expressed in lung T cells.

The observation that lung IL-17 production may play an important role in the immune containment of *M*. *tuberculosis* in the lung is consistent with several recent studies. The IL-17/IL-23 pathway is important for clearance of intracellular bacteria (83, 84), and it appears to be modulated during *M*. *tuberculosis* infection (85, 86). Protection of NHPs by pulmonary BCG vaccination was associated with lung TB-specific Th17 cells, and these were identified as the main correlate of protection (87). Interestingly, protection in this system was also associated with IL-10 production, which the authors speculate may derive from IL-10–producing Th17 cells, although this was not formally measured. In a separate NHP study, asymptomatic TB infection was associated with *M*. *tuberculosis*–specific IL-17–producing T cells in the BAL that were not detected in the blood (88). Moreover, lung-resident Th17 cells were identified as the key mediator of vaccine efficacy in a novel CysVac2/Advax mucosal TB vaccine of mice (67). In humans, high-dimensional profiling of blood from a large cohort of TB resistors and progressors in Peru revealed a persistent deficiency in polyfunctional Th17 cells in participants who went on to develop active disease (89). Therefore, tissue IL-17 responses are emerging as critical in the host immune response to *M*. *tuberculosis*. To investigate potential mechanisms, we tested whether exogenous IL-17 in the 3D granuloma model system affected cell survival, which may have an indirect effect on *M*. *tuberculosis* growth, or a more direct effect through the production of NO. IL-2 promoted cell survival in *M*. *tuberculosis*–infected microspheres, which may contribute the protective effect of this cytokine. This was not observed for IL-17, but both cytokines promoted NO production. NO is a key molecule in immune defense against *M*. *tuberculosis*, as shown in animal models using both chemical inhibition and genetic knockout of inducible NOS, the enzyme that produces NO (90). IL-17 as well as IL-2 have been shown to induce NO both in vitro and in vivo (91–94). Interestingly, NO was found to limit *M*. *tuberculosis* growth in mice by blocking IL-1β recruitment of granulocytes to the site of disease (95), suggesting a potential mechanistic relationship among IL-17, NO, and IL-1β. Whether these in vitro observations are important in vivo is not clear from these data, and further work is needed to understand the potential mechanisms.

Several important caveats to this study should be considered. All participants underwent surgical lung resection and therefore should be classified as having failed TB immunity. This is due to the nature of the study cohort and was unavoidable. However, data from NHPs demonstrate that both controlling and noncontrolling granuloma coexist in the same lung (72). In addition, before antibiotics, approximately 50% of participants with active TB eventually self-heal through their immune response (96). Therefore, it is likely that protective immune responses are present in the lungs of participants with progressive TB disease, even though they have failed to protect the whole organism. In this current study, multiple sections from different parts of the same lung were pooled to obtain sufficient cell numbers. In future studies, it will be informative to analyze different sections/granuloma from the same lung tissue to formally test whether TB adaptive immunity can vary locally. In addition, completely disease-free lung samples are simply unavailable, and we relied on non–TB-infected participants having surgery to remove lung tumors to generate control tissue. Although macroscopically healthy tissue was sampled, T cell phenotypes may be affected by the adjacent tumor mass. However, our phenotypic observations are in line with other recent work that studied human lung tissue (24). Finally, the use of plasma cytokines as a proxy marker of disease severity is imprecise and may relate to other sequelae, such as bronchiectasis. However, IL-1β was recently identified as the strongest cytokine marker of radiographic extent of TB disease, presence of large cavities, and TB smear grade in a clinical trial (57), indicating its potential use as a correlate of disease severity. In addition, the fact that these in vivo correlations suggesting an important role for IL-17 are backed up by our in vitro model, and by the recent publications highlighted above, strongly suggests that the observations are relevant.

Therefore, in conclusion, despite these limitations, we believe studies such as this, which characterize local tissue-resident immune responses, provide an invaluable resource in terms of understanding the human immune response toward *M*. *tuberculosis*. To the best of our knowledge, this is the first study in human TB to support a protective role for lung tissue-resident T cells producing IL-17. This adds strong support to growing evidence from animal models that tissue-resident Th17 cell responses are likely crucial to TB immunity and should be a key target for novel vaccine strategies.

## Methods

### Participants.

Whole blood was collected in tubes containing EDTA to prevent coagulation. Lung tissue was obtained from the King Dinizulu and Inkosi Albert Luthuli Central Hospitals in Durban, KwaZulu-Natal province, South Africa. Study participants included underwent surgically indicated pulmonary resections because of TB sequelae, which included bronchiectasis, hemoptysis, recurrent chest infections, and drug-resistant TB. All subjects undergoing surgery for TB sequelae were classified by the operating surgical team as having either suspected active TB or previous TB infection. This definition was based on clinical history and a review by the surgical team of preoperative chest x-ray and CT scans. Of 40 subjects with suspected active TB studied, 14 had matched histological information, of which 11 had indicative fibrocaseous or necrotizing granuloma and 2 had detectable acid-fast bacilli. Unless having emergency surgeries, all individuals undergoing lung surgery were placed on a minimum of 2 weeks of anti-TB drug therapy (usually Rifafour) to minimize risk to the operating surgeons, and thus, *M*. *tuberculosis* culture on tissue samples was not attempted. Control samples were obtained from macroscopically normal lung tissue from participants who underwent pulmonary resection for lung cancer. All participants provided informed consent and the study was approved by the Biomedical Research Ethics Committee.

### Sample preparation.

PBMCs were isolated from whole blood using Ficoll-Histopaque (MilliporeSigma) density gradient centrifugation and used fresh in assays or frozen in liquid nitrogen in freezing media (90% FBS containing 10% DMSO). Where frozen PBMCs were used, samples were thawed in DNase-containing (Roche, 25 U/mL) R10, which had been preheated to 37°C. Cells were rinsed and rested at 37°C overnight before assay setup.

Resected tissues were dissected and washed several times with cold HBSS (Lonza) before being resuspended in 8 mL of prewarmed digestion media R10 (RPMI supplemented with 10% FCS, 2 mM l-glutamate, 100 U/mL pen/strep), containing 0.5 mg/mL collagenase D (Roche) and 40 U/mL DNase I (Roche), and transferred to gentleMACS C tubes (Miltenyi Biotec) for mechanical digestion per manufacturer’s instructions. The resultant suspension was incubated for 30 minutes at 37°C, then subjected to an additional mechanical digestion step followed by another 30-minute incubation step at 37°C. The final suspension was strained through a 70 μm cell strainer and washed twice in HBSS. Following this step, cells were rested at 37°C overnight and lysed before counting in TC20 Automated Cell Counter (Bio-Rad) and assay setup.

### Mass cytometry staining and data analysis.

Lung cells were counted, stained in 100 μL containing APC-CD45 (1:20), and incubated on ice for 20 minutes. Cells were washed, then resuspended in 100 μL cold cyFACS buffer: PBS (Gibco, Thermo Fisher Scientific)+ 4% FBS + 0.05% sodium azide (MilliporeSigma). PBMCs were counted in 1 mL of cold cyFACS buffer and a maximum of 3 × 10^6^ live cells resuspended in 100 μL of cold cyFACS buffer. Both lung cells and PBMCs were kept on ice throughout the staining process. For participants with matched tissue and blood, 100 μL APC-CD45–positive lung cells were mixed with 100 μL of matched PBMCs. The total number of cells stained varied between 3 × 10^6^ and 5 × 10^6^. The samples were washed once, then resuspended in 100 μL of freshly diluted cisplatin (200 μM), for discrimination of live and dead cells, and incubated on ice for 5 minutes. Cells were washed and resuspended in 50 μL of cyFACS buffer containing surface primary antibody cocktail (ImmunoScape Pte Ltd) and incubated at 37°C for 15 minutes. After washing, cells were incubated with a secondary antibody cocktail (including the metal-labeled anti-fluorochrome antibody for the detection of live-cell barcoded tissue samples, ImmunoScape Pte Ltd) for 30 minutes on ice followed by fixing the cells in 2% paraformaldehyde in PBS overnight at 4°C. The next day the cells were washed, resuspended in freezing medium (90% FBS, 10% DMSO), and frozen later for CyTOF acquisition. After thawing, cells were further stained for panel-specific intracellular markers and DNA, and each sample was barcoded with a unique combination of 2 distinct cellular barcodes (97). Cells were washed and adjusted to 0.5 million cells/mL H_2_O together with 1% equilibration beads (EQ Four Element Calibration Beads, Fluidigm) for acquisition on a CyTOF Helios system (Fluidigm).

Signals for each parameter were normalized based on EQ beads added to each sample. Any 0 values were randomized using a custom R script that uniformly distributes values between –1 and 0. Each sample was manually debarcoded followed by gating on DNA^+^ cells. Immune cells were identified by gating on live (cisplatin^–^) CD45^+^ cells, and tissue and PBMCs from the same donor were further identified according to the tissue-specific live-cell barcode tag (APC-CD45). Subset identification followed a gating cascade according to the lineage markers using FlowJo (Tree Star) software. High-dimensional data analysis was performed using ImmunoScape’s cloud-based analytical pipeline tool, Cytographer. For the visualization of high-dimensionality data, UMAP as a dimensionality reduction technique (98) was used. Phenotypic dissection was performed using the PhenoGraph clustering algorithm (99). Marker expression intensities were represented as heatmaps and expression plots. Dot plots and UMAP plots were displayed using FlowJo.

### Intracellular cytokine and transcription factor staining.

Lung cells and PBMCs were plated in each well of a round-bottom, 96-well plate and incubated in the presence of MTB300 peptide (2 μg/mL, La Jolla Institute for Immunology). Incubation in the presence of a mixture of PMA (0.5 μL/200 μL) and ionomycin (0.3 μL/200 μL) or in the absence of stimuli were used as positive and negative controls, respectively. Cells were incubated for 1 hour at 37°C, at which point GolgiStop and GolgiPlug (BD Biosciences) were added to each well for the remaining 4 hours.

### Flow cytometry.

For all experiments, identification of immune cells was done by surface staining with a near-infrared live/dead cell viability cell staining kit (Invitrogen, Thermo Fisher Scientific) and a combination of the following fluorochrome-conjugated antibodies: αCD45-V500 Horizon clone HI30 (BD Biosciences), αCD3 Brilliant Violet 785 clone OKT3 (BioLegend), αCD4 Brilliant UltraViolet 496 clone SK3 (BD Biosciences), αCD8 Brilliant Violet 605 clone RPA-T8 (BioLegend), αCD19-FITC clone HIB19 (BD Biosciences), αCD62L-PE-Cy5 clone DREG-56 (BD Biosciences), αCD103-APC clone Ber-ACT8 (BD Biosciences), αCD45RA Brilliant Violet 650 clone HI100 (BD Biosciences), αCCR7 PerCP-Cy5.5 clone G043H7 (BioLegend), αCD25 Brilliant Violet 711 clone BC96 (BioLegend), αCD56 Brilliant Violet 711 clone HCD56 (BioLegend), αCD16 Brilliant Violet 650 clone 3G8 (BioLegend), αCD69 Brilliant UltraViolet 395 clone FN50 (BD Biosciences), αPD-1 Brilliant Violet 421 clone EH12.1 (BD Biosciences), αCTLA-4 PE L3D10 (BioLegend), and αTIM-3 Alexa Fluor 700 clone 344823 (R&D Systems, Bio-Techne). Cells were surface stained with 25 μL of antibody cocktail in the dark for 20 minutes at room temperature followed by washing with PBS. Where intracellular staining was not performed, cells were immediately fixed with 2% paraformaldehyde, then acquired on FACSAria Fusion (BD Biosciences).

For intracellular cytokine staining, surface stain was washed off, and cells were permeabilized using Fix/Perm kit (BD Biosciences) for 20 minutes at 4°C and washed. Then 20% goat serum was added for 20 minutes at room temperature in the dark to block nonspecific antibody binding. Following a washing step, 25 μL of the following cytokine cocktail was added: anti–TNF-α Alexa Fluor 700 clone MAb11 (BD Biosciences), anti–IL-2 PE-CF594 clone 5344.111 (BD Biosciences), anti–IFN-γ PE-Cy7 clone 4S.B3 (BD Biosciences) or Brilliant Violet 421 clone 4S.B3 (BioLegend), and anti–IL-17 PE clone BL168 (BioLegend).

For measurement of transcription factor FoxP3, the eBioscience fixation/permeabilization kit (Thermo Fisher Scientific) was used for intracellular staining then blocked with 20% goat serum for 20 minutes prior to antibody staining with FoxP3 eFlour 450 clone PCH101 (eBioscience, Thermo Fisher Scientific). Data acquisition was performed using a FACSAria Fusion or FACSAria III cytometer (BD Biosciences) and analyzed using FlowJo v.9.9.

### Plasma cytokine analysis.

Plasma samples were collected from whole blood and frozen at –80°C until needed. TNF-α, IL-17A, and IL-1β levels were quantified using multiplex high-sensitivity MILLIPLEX MAP kits (MilliporeSigma) on a Bio-Plex 200 system (Bio-Rad) following manufacturers’ instructions.

### Cell encapsulation to form 3D culture microspheres.

Microspheres were generated as previously described (58, 60). PBMCs were isolated using density gradient centrifugation over Ficoll-Paque (GE Healthcare Life Sciences, now Cytiva) from apparently healthy blood donors (ethical approval ref. 13/SC/0043). Bioluminescent *M*. *tuberculosis* H37Rv was routinely cultured in Middlebrook 7H9 medium (BD Biosciences) supplemented with 10% ADC enrichment (Scientific Laboratory Supplies), 0.2% glycerol, and 0.02% Tween 80 with kanamycin (25 μg/mL). For all experiments, bacterial cultures were grown to optical density of 0.6 (~1 × 10^8^ CFU/mL). Host cells were then infected with luminescent mycobacteria at a multiplicity of infection of 0.1. After overnight incubation at 37°C in 5% CO_2_, the infected PBMCs were treated with Versene solution for 10 minutes and neutralized by HBSS without Ca/Mg (both from Gibco, Thermo Fisher Scientific). The cells were detached by scraping, placed in 50 mL Falcon tubes (Corning), topped up with HBSS, and spun at 320*g* for 8 minutes at 4°C. To obtain a 3D culture, we resuspended *M*. *tuberculosis*–infected host cells in sterile alginate-collagen matrix at 5 × 10^6^ cells/mL. The mix was injected into the electrostatic bead generator (Nisco) to form microspheres via a Harvard syringe driver as described previously (100). After washing 2 times in HBSS without Ca/Mg, microspheres were equally distributed into 2 mL Eppendorf tubes, immersed in 1 mL RPMI medium (consisting of RPMI 1640 medium supplemented with 10 μg/mL of ampicillin, 2 mM of glutamine, 25 μg/mL of kanamycin, and 10% of human AB serum) with or without cytokines, and incubated at 37°C and 5% CO_2_. We monitored bacterial bioluminescence using a GloMax 20/20 Luminometer (Promega). All the cytokines were purchased from ImmunoTools, suspended in RPMI with 0.1% human AB serum, and kept at –80°C until use. The cytokines included IL-2, IL-17A, TNF-α, and IFN-γ. A total of 250 μL of media were taken and replaced with an equal amount of the media with or without 4 times of the cytokine concentration. Bacterial growth was monitored with luminescence using GloMax Discover microplate reader (Promega). Cell viability in microspheres was determined by LDH release in the supernatants collected at day 7. This was analyzed by a colorimetric activity assay as per manufacturer’s instructions (Roche). LDH was quantified using LDH standards provided, and results were normalized using levels in microspheres with no cytokine in each experiment. NO production in microspheres was determined by detection of accumulated nitrites (NO_2_^−^) in the cell supernatants using the Griess reagent system (Promega) according to the manufacturer’s instructions. Briefly, 100 μL of the cell supernatant was incubated with 100 μL Griess reagent (Promega) for 15 minutes in the dark at room temperature, and the absorbance was measured at 546 nm on a GloMax Discover UV/Vis microplate reader (Promega). The concentrations of nitrites were derived by regression analysis using serial dilutions of sodium nitrite as a standard and normalized with the amount from microspheres with no cytokine for each experiment.

### Statistics.

All statistical analyses were performed using GraphPad Prism version 6.0d (GraphPad Software, Inc). Comparisons of 2 groups were done by a paired or unpaired 2-tailed Student’s *t* test (Wilcoxon’s test or Mann-Whitney test, respectively), where a *P* value of 0.05 and below was statistically significant. Significance of more than 2 groups was determined using a Kruskal-Wallis test, or where specific comparisons between 2 or more groups were relevant, a Mann-Whitney test with Bonferroni’s correction for multiple comparison was done. *P* values of *P* < 0.05 are shown, with values that passed correction for multiple comparisons highlighted with asterisks.

### Study approval.

All participants provided informed consent, and the study was approved by the Biomedical Research Ethics Committee of the University of KwaZulu-Natal (BE019/13). Apparently healthy blood donor study ethical approval was provided by the National Research Ethics Service Committee South Central — Southampton A, ref 13/SC/0043, Southampton, United Kingdom.

## Author contributions

PO conducted all flow experiments and assisted with data analysis and writing of the manuscript; LBT carried out all 3D model experiments; AA, SN, and A Ng’oepe conducted CyTOF studies and assisted in sample preparation; MF, BHL, A Nardin, FK, KK, KJD, and RM obtained samples and analyzed clinical information; CSLA and A Sette provided MTB300; AJCS established the lung cohort; DR and A Singh assisted in sample preparation; TN obtained samples and analyzed clinical information; SMB helped with data interpretation and manuscript preparation; HNK and PTE helped with data interpretation, study design, and manuscript preparation; and AL is the senior author who designed and implemented this study, analyzed the data, and cowrote the manuscript with PO.

## Supplementary Material

Supplemental data

## Version 1 04/13/2021

In-Press Preview

## Version 2 05/17/2021

Electronic publication
