# Supplementary material for: Tissue-resident-like CD4+ T cells secreting IL-17 control Mycobacterium tuberculosis in the human lung
Source: J Clin Invest. 2021 May 17;131(10):e142014. doi: 10.1172/JCI142014 (PMC8121523; doi:10.1172/JCI142014)
Supplement: Supplemental data [file jci-131-142014-s066.pdf]

Supplementary figures

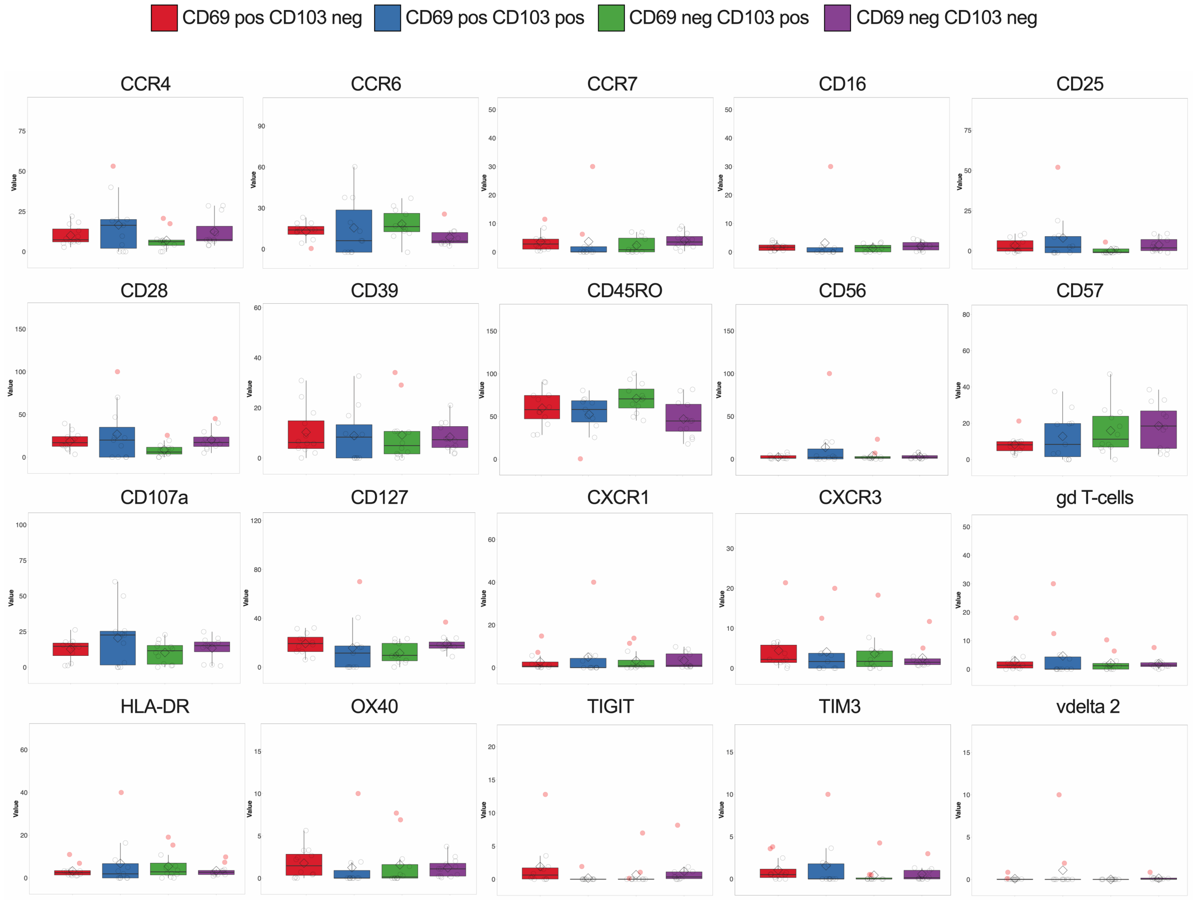

Supplementary Figure 1. Identification of tissue-resident CD4 T-cells in lung and blood from study participants. Expression pattern of surface markers measured by CyTOF not significantly differential expressed in lung homogenate between cells expressing combination of CD69 and/or CD103.

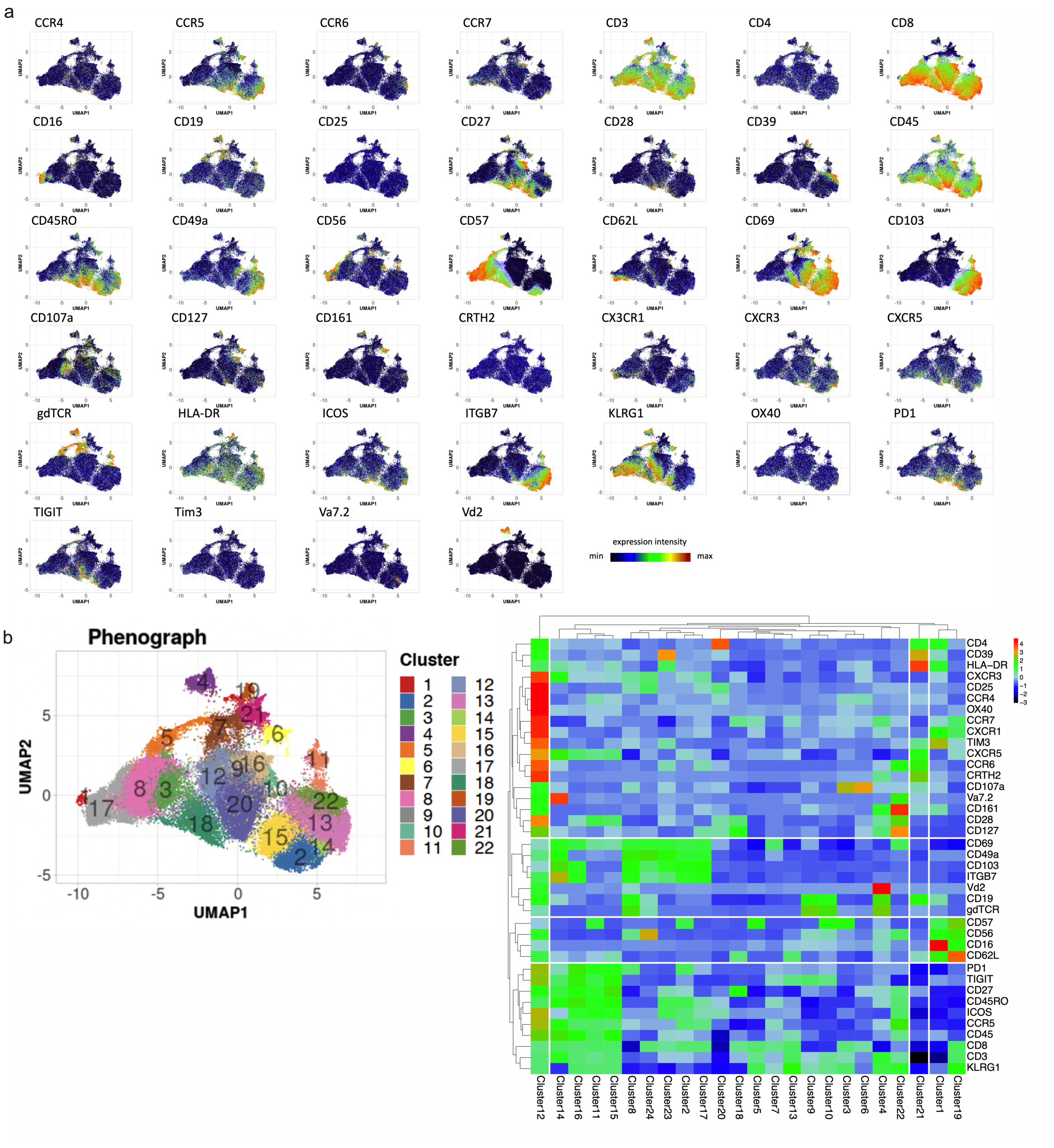

Supplementary Figure 2. Identification of tissue-resident CD8 T-cells in lung and blood from study participants. (a) Cumulative staining of lung CD8 T-cells from 12 biological replicates, defined as having either active TB or previous TB by CyTOF high dimensional phenotyping based on UMAP plotted as UMAP1 (x-axis) vs UMAP2 (y-axis) for each cell type. (**b**) Phenograph clustering (left) identified 22 clusters (cluster 1-22) depicted on the heatmap of staining intensity of T-cell markers (right).

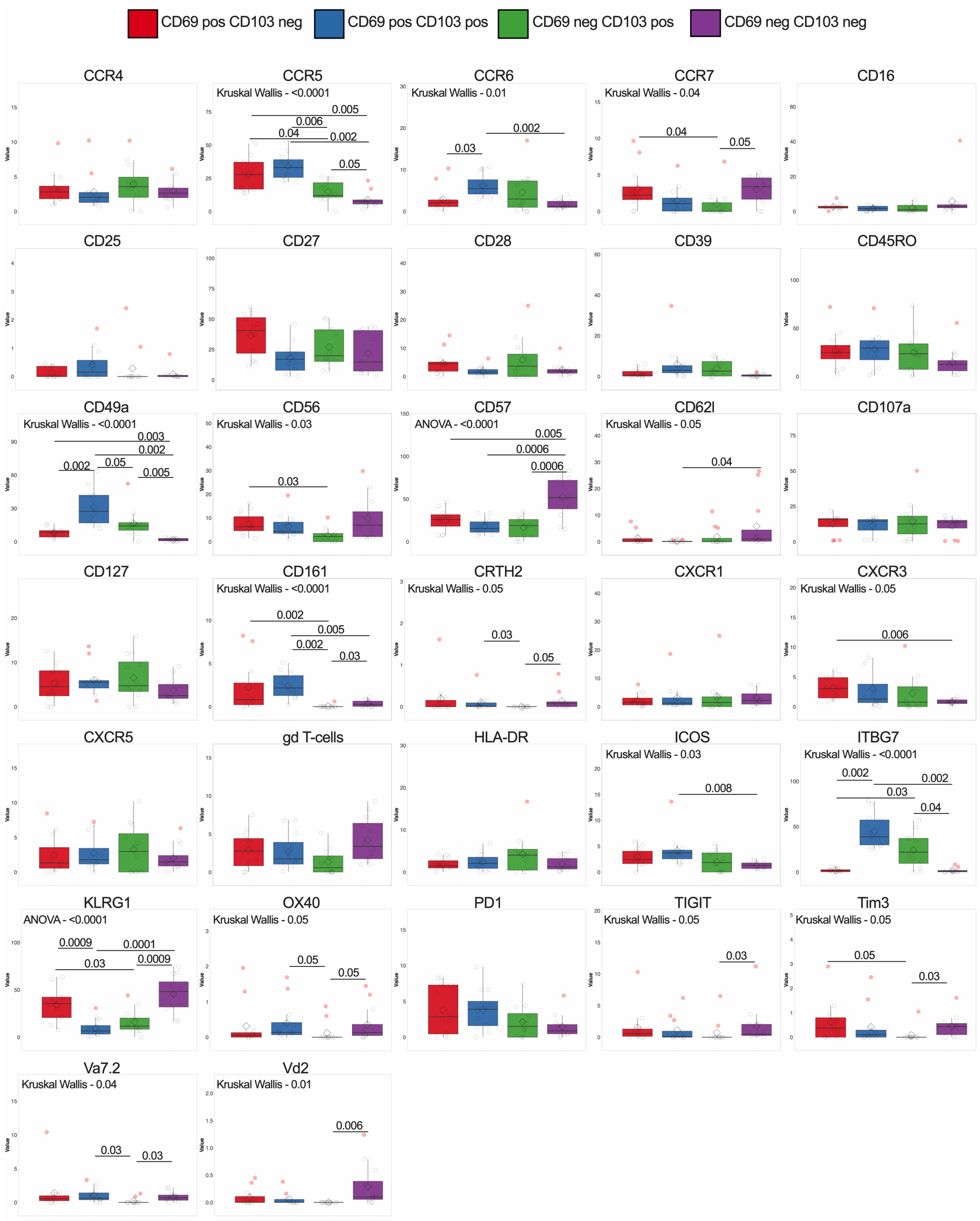

Supplementary Figure 3. Identification of tissue-resident CD8 T-cells in lung and blood from study participants. Expression pattern of surface markers measured by CyTOF on CD8 T-cells in lung homogenate between cells expressing combination of CD69 and/or CD103. Significance testing indicated for each panel.

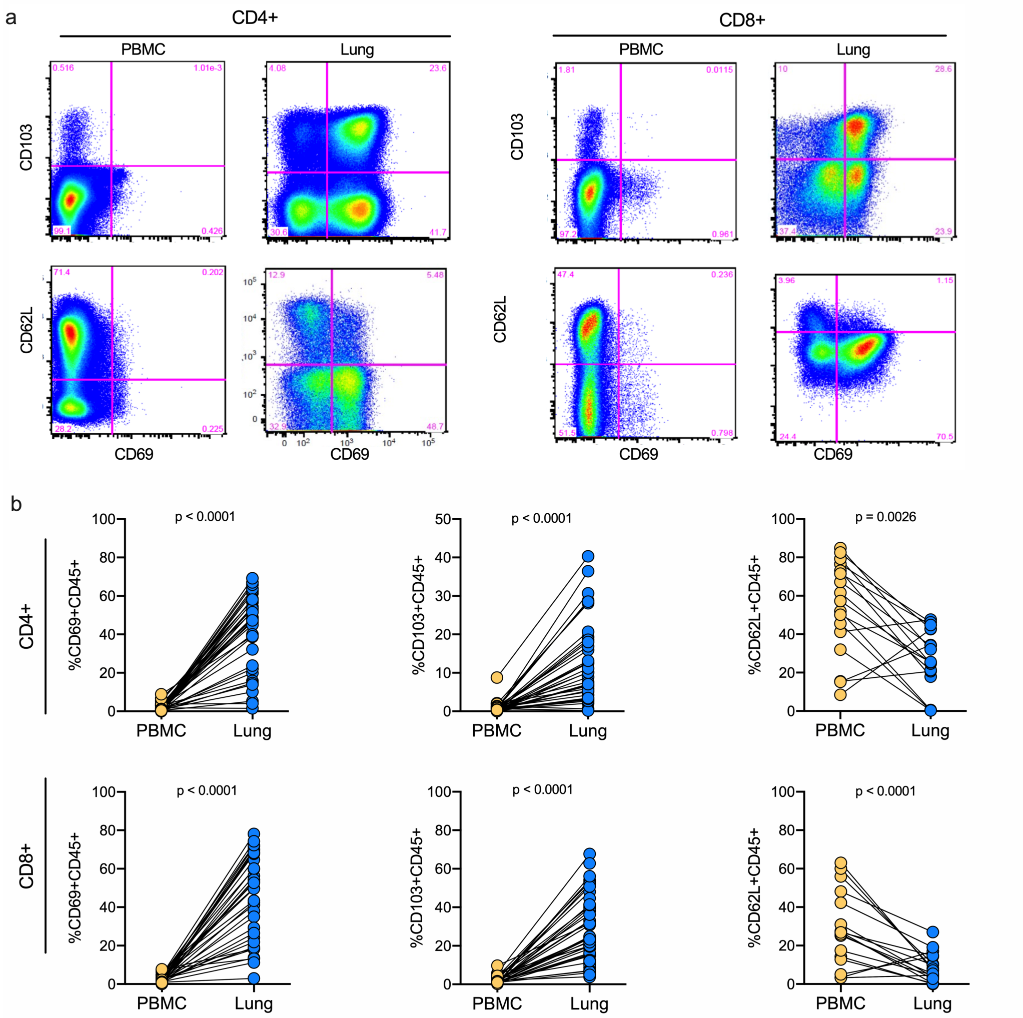

Supplementary Figure 4. Identification of tissue-resident T-cells in lung and blood from study participants by conventional flow cytometry (a) Representative flow cytometry plots showing expression of tissue-residence (CD69 and CD103) and blood (CD62L) markers in $CD4^+$ and $CD8^+$ T-cells from blood and lung of a participant with active TB. (**b**) Cumulative frequencies of CD69, CD103 and CD62L expressing CD4+ and CD8+T-cells from paired blood (yellow) and lung (blue) samples from the same participants. Significance by Wilcoxon matched-pairs signed rank test.

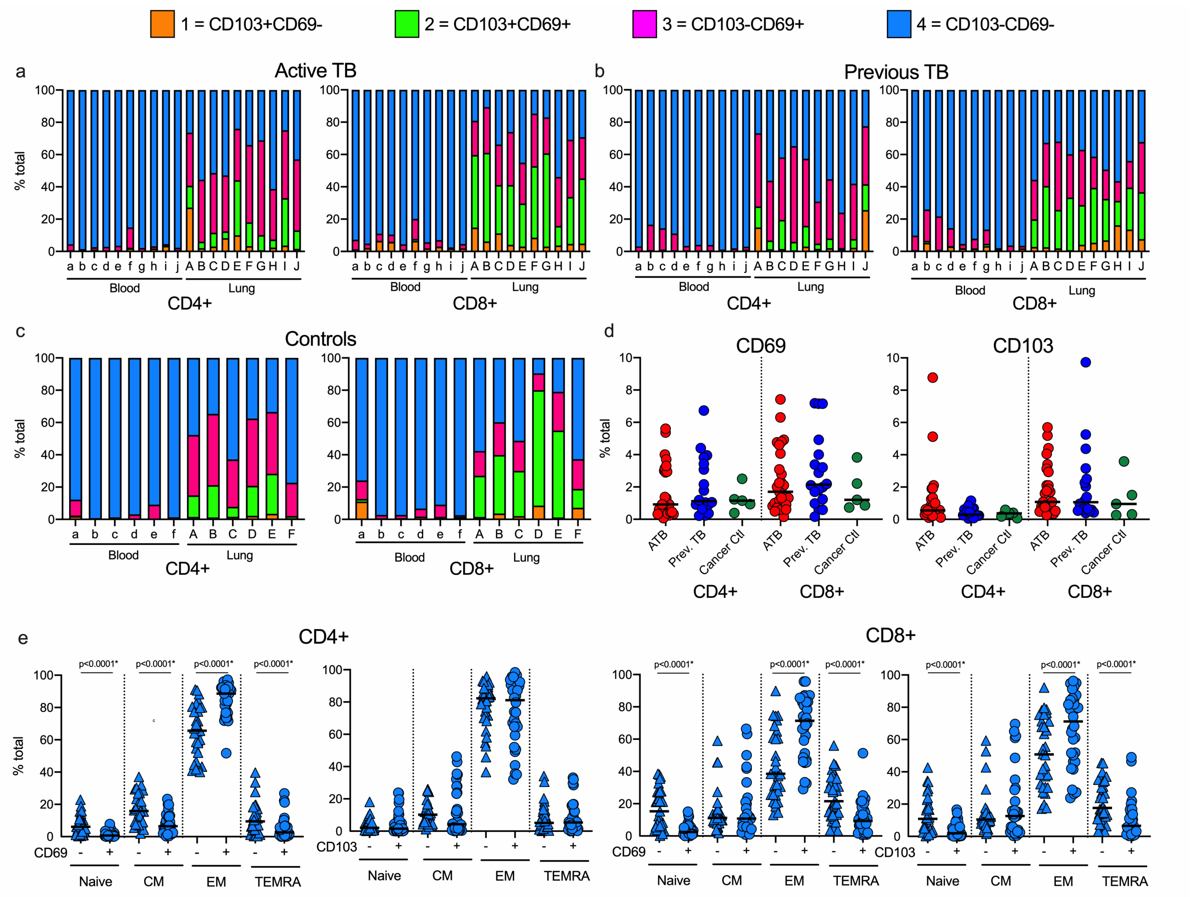

Supplementary Figure 5. Tissue-residence and effector memory phenotypes of T-cells isolated from human lung tissues. (**a-c**) Tissue-resident phenotypes of CD4+ (left) and CD8+ (right) T-cells from blood (a-j) and lung tissue (A-J) from participants with active TB (a), previous TB (b) and cancer control blood (a-f) and lung tissue (A-F) (c) where orange = CD103$^+$CD69$^-$, green = CD103$^-$CD69$^+$, pink = CD103$^+$CD69$^+$ and blue = CD103$^-$CD69$^-$. (**d**) Frequencies of CD69$^+$ CD4 and CD8 T cells (left) and CD103$^+$ CD4 and CD8 T cells (right) in peripheral blood from participants with active TB (red), previous TB (dark blue) or cancer controls (dark green). Significance calculated by Mann-Whitney test with Bonferroni corrections, although none was found. Significance calculated by Mann-Whitney test. * denotes p-values which remained significance after stringent Bonferroni correction for multiple comparisons. (**e**) Frequencies of CD69- or CD103- (triangles) and CD69+ or CD103+ (circles) CD4+ (top panels) and CD8+ (bottom panels) T-cells from lung that are naïve or display central memory (CM), effector memory (EM) or terminally differentiated (TEMRA) phenotype. Significance calculated by Mann-Whitney test. * denotes p-values which remained significance after stringent Bonferroni correction for multiple comparisons, median values indicated with a black line.

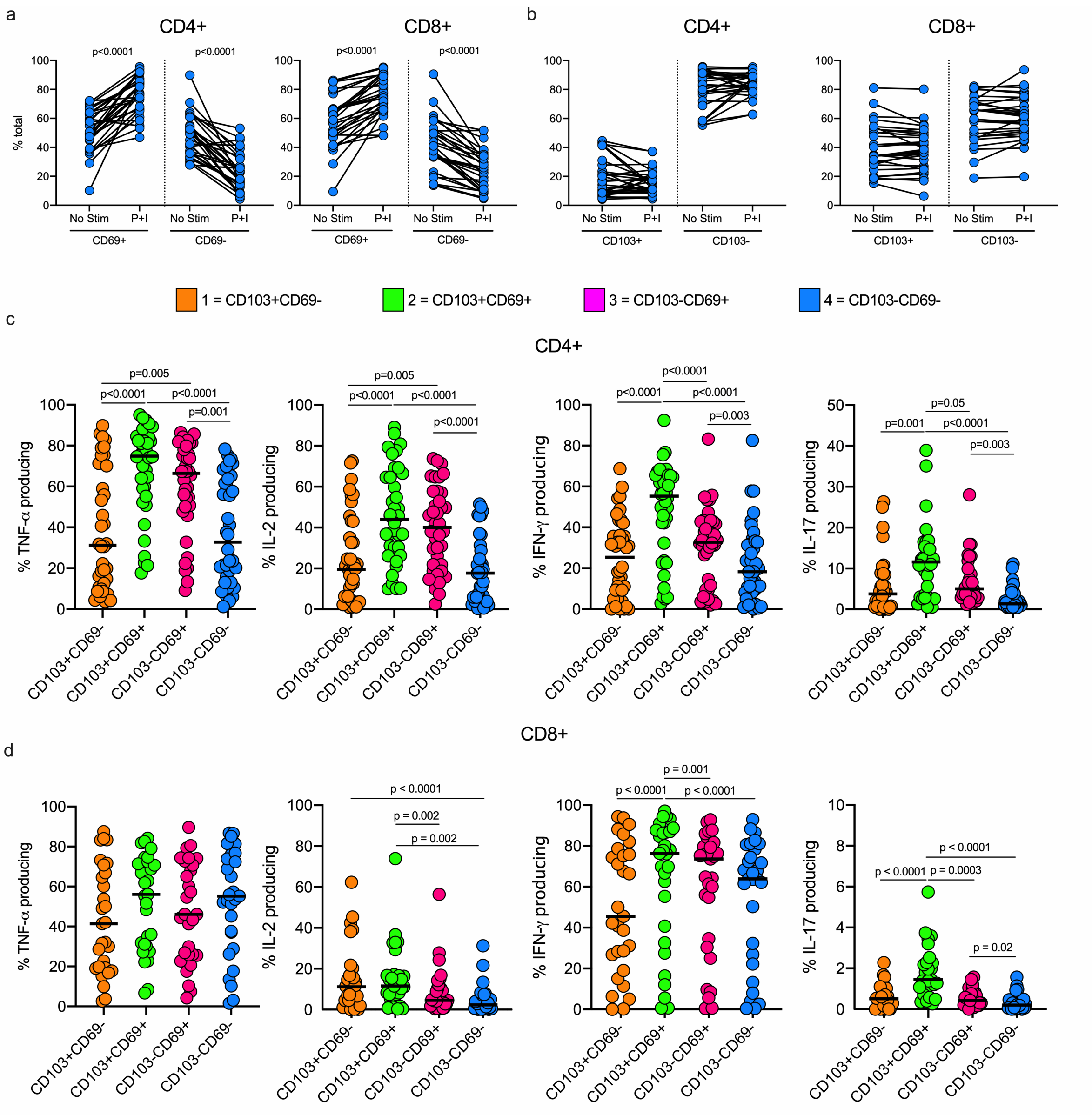

Supplementary Figure 6. Tissue-resident T-cells are capable of prolific cytokine production. (**a**) Stimulation with PMA and Ionomycin (P+I) is associated with increased CD69 expression in lung $CD4^+$ (left) and $CD8^+$ (right) T-cells. Significance by Wilcoxon matched-pairs signed rank test. (**b**) Stimulation with PMA and Ionomycin (P+I) is not associated with increased CD103 expression in lung $CD4^+$ (left) and $CD8^+$ (right) T-cells. Significance by Wilcoxon matched-pairs signed rank test. (**c-d**) Tissue-resident phenotypes of TNF-α, IL-2, IFN-γ and IL-17 producing $CD4^+$ (**c**) and $CD8^+$ (**d**) T-cells from lung where orange = $CD103^+CD69^-$, green = $CD103^-CD69^+$, pink = $CD103^+CD69^+$ and blue = $CD103^-CD69^-$. Significance calculated by Kruskal-Wallis test, median values indicated with a black line.

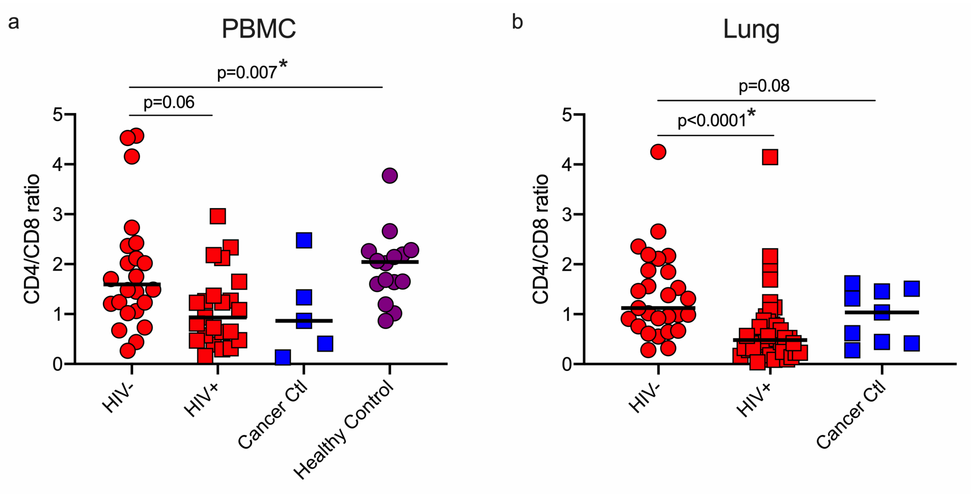

Supplementary Figure 7. HIV is associated with significantly reduced CD4/CD8 ratios in blood and lung. (**a**) CD4/CD8 ratios in blood from active TB/previous TB participants with (red squares) and without (red circles) HIV co-infection, cancer controls (dark blue) and healthy controls (purple). (**b**) CD4/CD8 ratios in lung tissues from active TB/previous TB participants with (red squares) and without (red circles) HIV co-infection and cancer controls (dark blue). Significance calculated by Mann-Whitney test. * denotes p-values which remained significance after stringent Bonferroni correction for multiple comparisons, median values indicated with a black line.

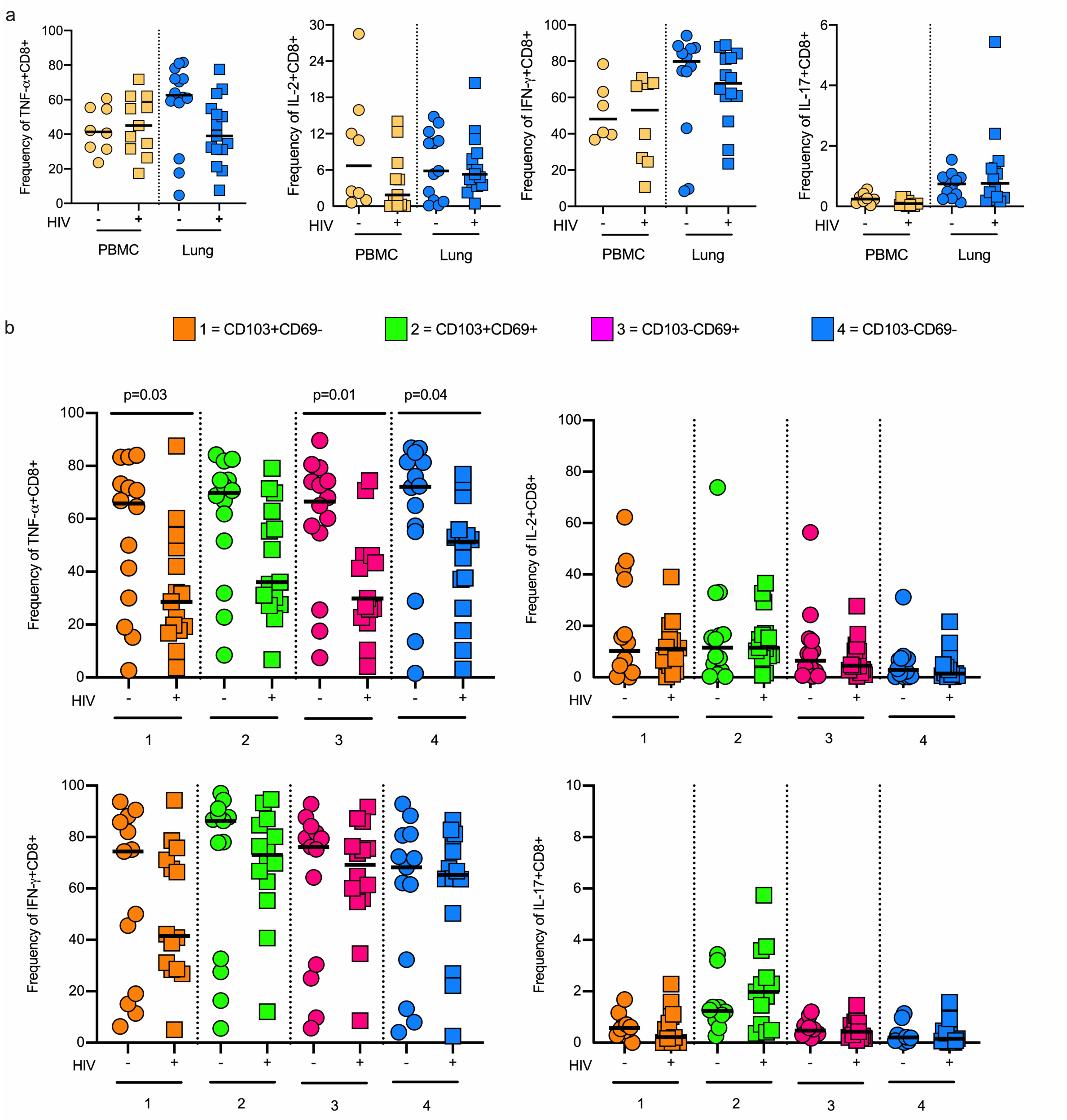

Supplementary Figure 8. HIV depletes cytokine-producing Trms from the lungs of TB-infected study participants. (**a**) Frequencies of TNF-α, IL-2, IFN-γ and IL-17 producing CD8$^+$T-cells from blood (yellow) and lung (blue) of participants with (squares) and without (circles) HIV co-infection. (**b**) Tissue-resident phenotypes of TNF-α, IL-2, IFN-γ and IL-17 producing CD8$^+$T-cells from lung from participants with (squares) and without (circles) HIV co-infections, where 1 (orange) = CD103$^+$CD69$^-$, 2 (green) = CD103$^-$CD69$^+$, 3 (pink) = CD103$^+$CD69$^+$ and 4 (blue) = CD103$^-$CD69$^-$. Significance calculated by Mann-Whitney test, median values indicated with a black line.

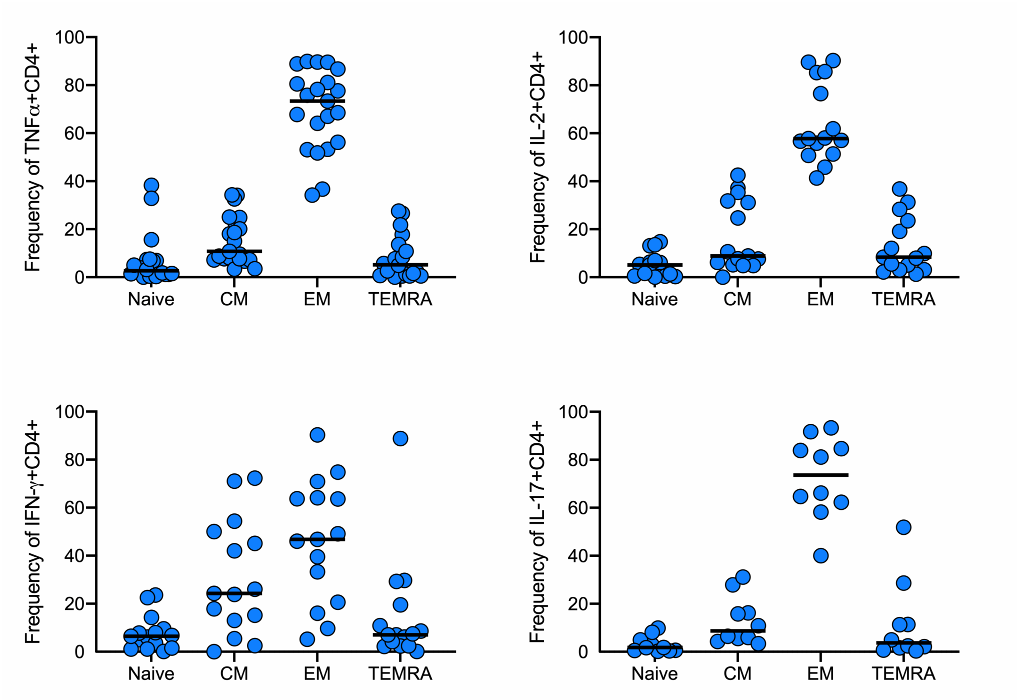

Supplementary Figure 9. TB-specific CD4+T-cells are predominantly effector memory cells. Frequencies of MTB300-specific TNF-α, IL-2, IFN-γ and IL-17 producing $CD4^{+}$T-cells in lung tissues were assessed for the expression of CD45RA and CCR7. Participants with active/previous TB combined express various levels of naïve, central memory (CM), effector memory (EM) or terminally differentiated (TEMRA) phenotype. Median values indicated with a black line.

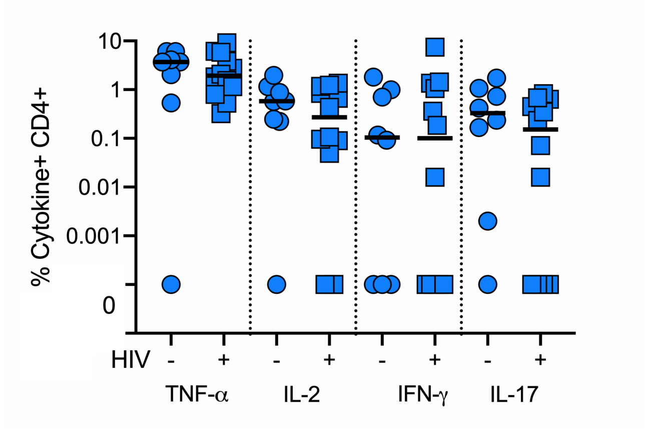

Supplementary Figure 10. HIV does not affect cytokine production by TB-specific, lung-resident $CD4^{+}$T-cells. Frequencies of TNF-α, IL-2, IFN-γ and IL-17 producing $CD4^{+}$T-cells in lung tissues from participants with (squares) and without (circles) HIV co-infection. Significance tested by Mann-Whitney test with Bonferroni corrections, although none was found, median values indicated with a black line.

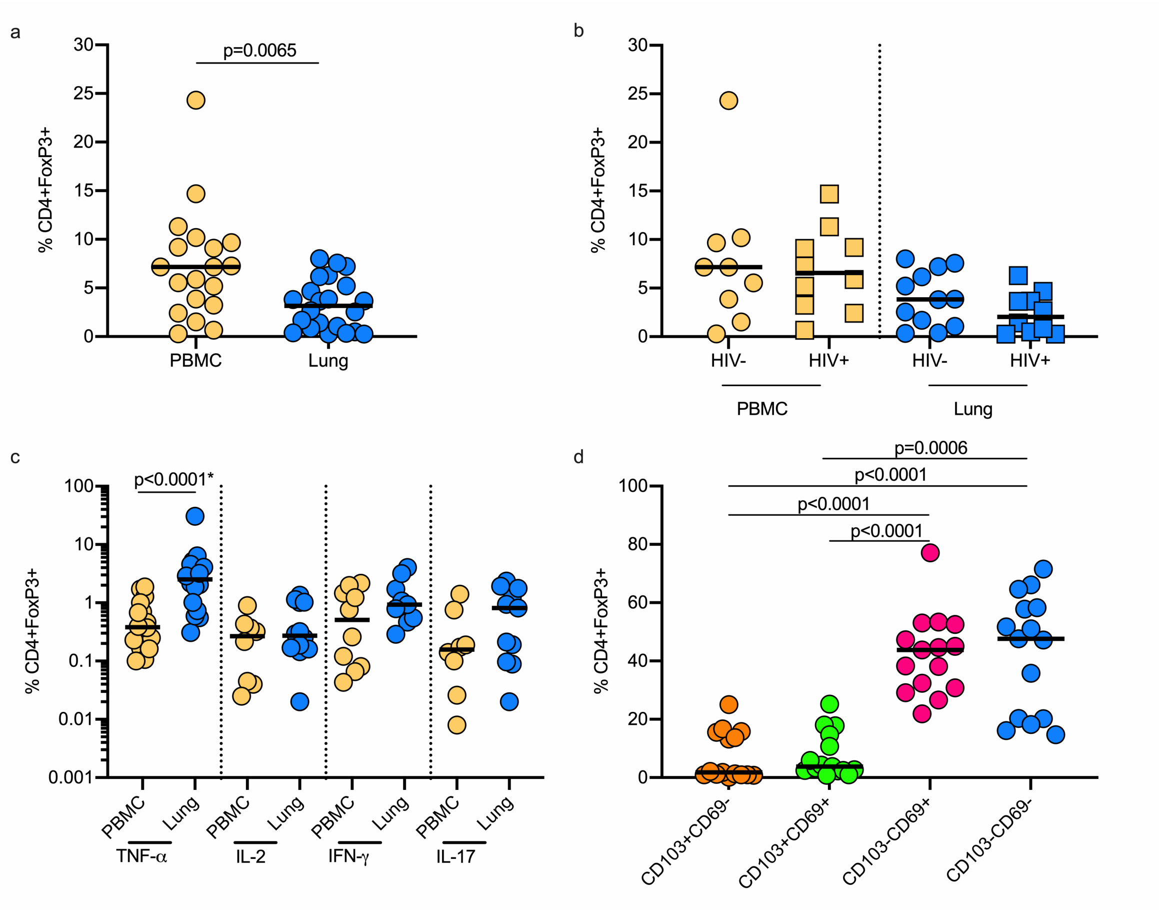

Supplementary Figure 11. The lung contains populations of Tregs that are not affected by HIV co-infection. (**a**) Frequencies of FoxP3+ Tregs in blood (yellow) and lung tissue (blue) from participants with active/previous TB. Significance by Mann-Whitney test. (**b**) Frequencies of FoxP3+ Tregs in blood (yellow) and lung tissue (blue) from participants with (squares) and without (circles) HIV co-infection. (**c**) Frequencies of CD4$^+$FoxP3$^+$T-cells that produce TNF-α, IL-2, IFN-γ and IL-17 in blood (yellow) or lung (blue) from participants with active/previous TB. Significance by Mann-Whitney test. * denotes p-values which remained significance after stringent Bonferroni correction for multiple comparisons. (**d**) Tissue-residence phenotypes of FoxP3+ Tregs where orange = CD103$^+$CD69$^-$, green = CD103$^-$CD69$^+$, pink = CD103$^+$CD69$^+$ and blue = CD103$^-$CD69$^-$. Significance by Mann-Whitney test. * denotes p-values which remained significance after stringent Bonferroni correction for multiple comparisons. In all cases median values indicated with a black line.

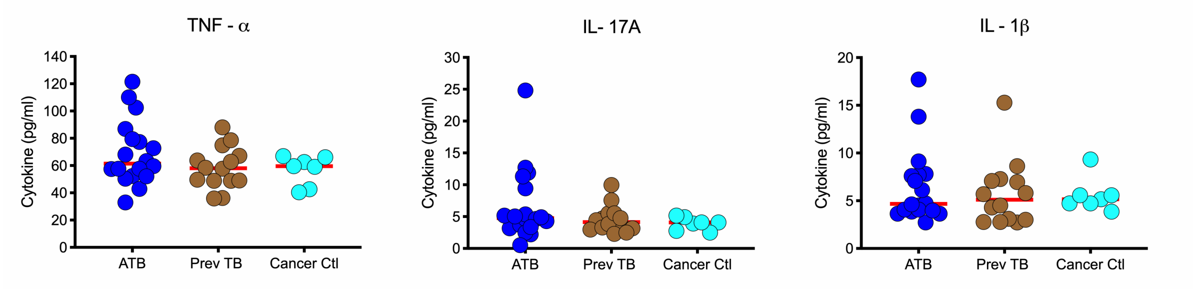

Supplementary Figure 12. Plasma markers of systemic inflammation not elevated in TB infected participants. TNF-α, IL-17A and IL-1β was measured in plasma of active TB (blue), previous TB (brown) and cancer control (cyan). In both active and previous TB groups, there were some participants whose cytokine levels were higher than that of control participants. However, there was no significant difference in the mean level of cytokines in the groups measured. Significance tested by Mann-Whitney test with Bonferroni corrections, although none was found. In all cases median values indicated with a red line.

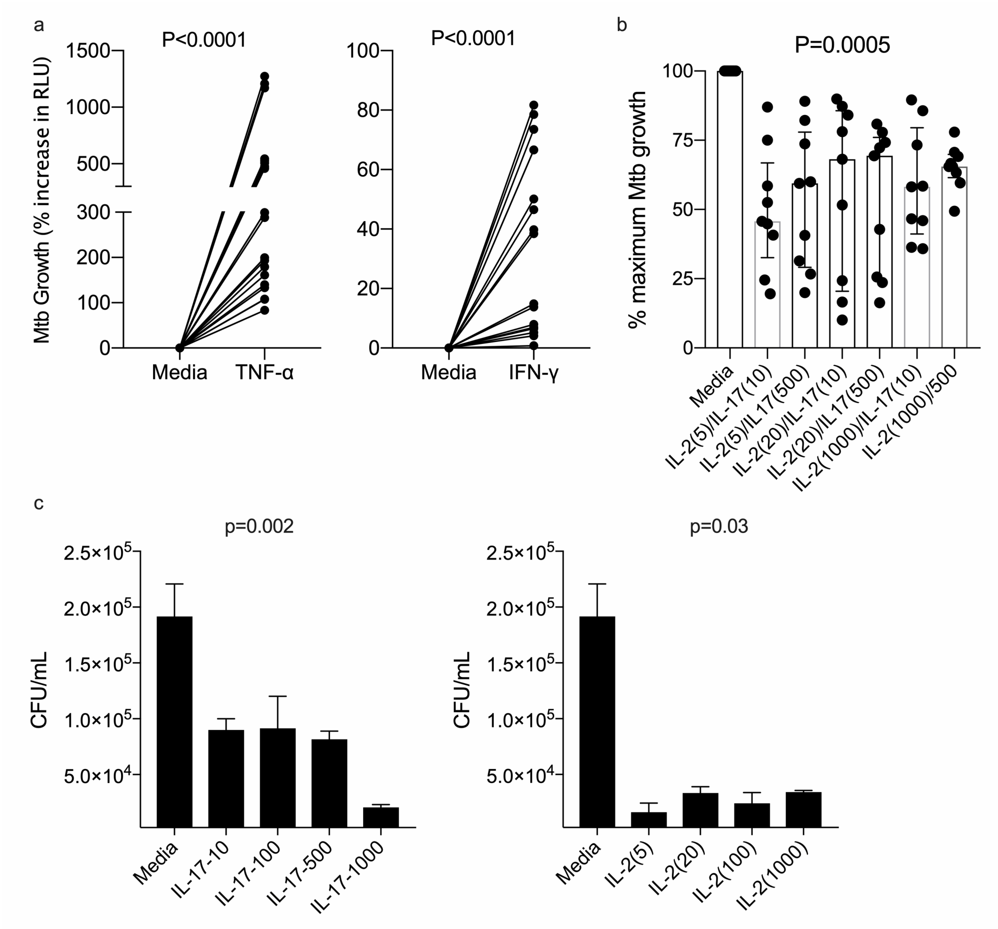

Supplementary Figure 13a. effect of exogenous cytokines on Mtb growth in 3D model. (a) Addition of exogenous TNF-α or IFN-γ significantly increases growth of luminescent Mtb in 3D model system. Cumulative data from 5 separate experiments using PBMC from 5 separate health donors, run in triplicate; compare by Mann-Whitney paired T-cells. (b) No synergistic effect observed when IL-17 and IL-2 given in combination measured. Significance calculated by Mann-Whitney test. (c) suppressive effect of exogenous IL-17 and IL-2 confirmed by CFU on day 15, significance tested my Kruskal-Wallis. Bars indicate median values with interquartile ranges.
